# Switching Between Reference Biologics and Biosimilars for the Treatment of Rheumatology, Gastroenterology, and Dermatology Inflammatory Conditions: Considerations for the Clinician

**DOI:** 10.1007/s11926-017-0658-4

**Published:** 2017-06-16

**Authors:** Robert Moots, Valderilio Azevedo, Javier L. Coindreau, Thomas Dörner, Ehab Mahgoub, Eduardo Mysler, Morton Scheinberg, Lisa Marshall

**Affiliations:** 1 University of Liverpool, Liverpool, UK; 2 Department of Musculoskeletal Biology, Institute of Ageing and Chronic Disease, Clinical Sciences Centre, Aintree University Hospital, Longmoor Lane, Liverpool, L9 7AL UK; 3 Federal University of Parana and Edumed Health Research Center and Biotech, Curitiba, Brazil; 4 Pfizer Inc, Collegeville, PA USA; 5 Department of Medicine, Rheumatology and Clinical Immunology, Charité Universitätsmedizin Berlin, Berlin, Germany; 6 Organización Médica de Investigación, Buenos Aires, Argentina; 7 Hospital Israelita Albert Einstein, São Paulo, Brazil

**Keywords:** Biologics, Biosimilars, Switching, Clinical trials, Real world data

## Abstract

**Purpose of Review:**

Biosimilars of the reference biologic therapeutics infliximab, etanercept, adalimumab, and rituximab are entering the market. Clinical and real-world data on the effects of reference → biosimilar switching are limited. This review was carried out to assess the current body of switching data.

**Recent Findings:**

Fifty-three switching studies were identified. Infliximab publications covered CT-P13 (25 studies), SB2 (1), infliximab NK (1), and unspecified infliximab biosimilars (2). Etanercept publications covered SB4 (2) and GP2015 (2). Adalimumab publications covered ABP 501 (2) and SB5 (1). Rituximab publications covered CT-P10 (1). Efficacy and safety data generally showed no differences between patients who switched treatments versus those who did not. No differences were seen pre- and post-switch. Immunogenicity data were presented in 19/37 (51%) studies.

**Summary:**

Additional data from switching studies of these therapies are still required, as is continuing pharma-covigilance. Switching should remain a case-by-case clinical decision made by the physician and patient on an individual basis supported by scientific evidence.

**Electronic supplementary material:**

The online version of this article (doi:10.1007/s11926-017-0658-4) contains supplementary material, which is available to authorized users.

## Introduction

Biosimilars are biologic products assessed by regulatory agencies to be similar to a licensed reference product in terms of quality, safety, and efficacy. Different agencies have their own definitions of biosimilarity [1–3], and regional regulatory requirements for biosimilars have been discussed elsewhere [4]. Proposed biosimilar products include both candidate biosimilars (copies of licensed reference products still in development) and intended copies (products marketed without first undergoing rigorous comparative evaluations) [5]. The development of proposed biosimilar products has increased as reference drugs lose patent exclusivity, with the anticipated effect of increasing patient access through reduced costs.

A key question for health care professionals (HCPs) contemplating prescribing biosimilar drugs is “Should the biosimilar immediately replace the reference product currently in use by the stable patient?” When considering this, HCPs should take into account not only the efficacy and safety of the biosimilar, but also any possible effects of switching patients from the reference to its biosimilar product. Such effects can be identified by clinical and real-world studies of switching. With each approach, the goal is to demonstrate no loss of efficacy or increase in safety risk when transitioning patients between the two compounds. However, regulatory agencies generally do not require switching studies in order to approve a biosimilar, resulting in registration studies that do not always assess the effects of switching patients between treatments. The United States Food and Drug Administration (US FDA) is an exception to this, requiring a single transition evaluation to demonstrate that a biosimilar can be switched with a reference product [6], and a study with three reference → biosimilar switches to demonstrate interchangeability [7]. When switching data are unavailable to inform clinical decisions, this may negatively impact the HCP’s ability to offer optimal treatment.

**Fig. 1:**
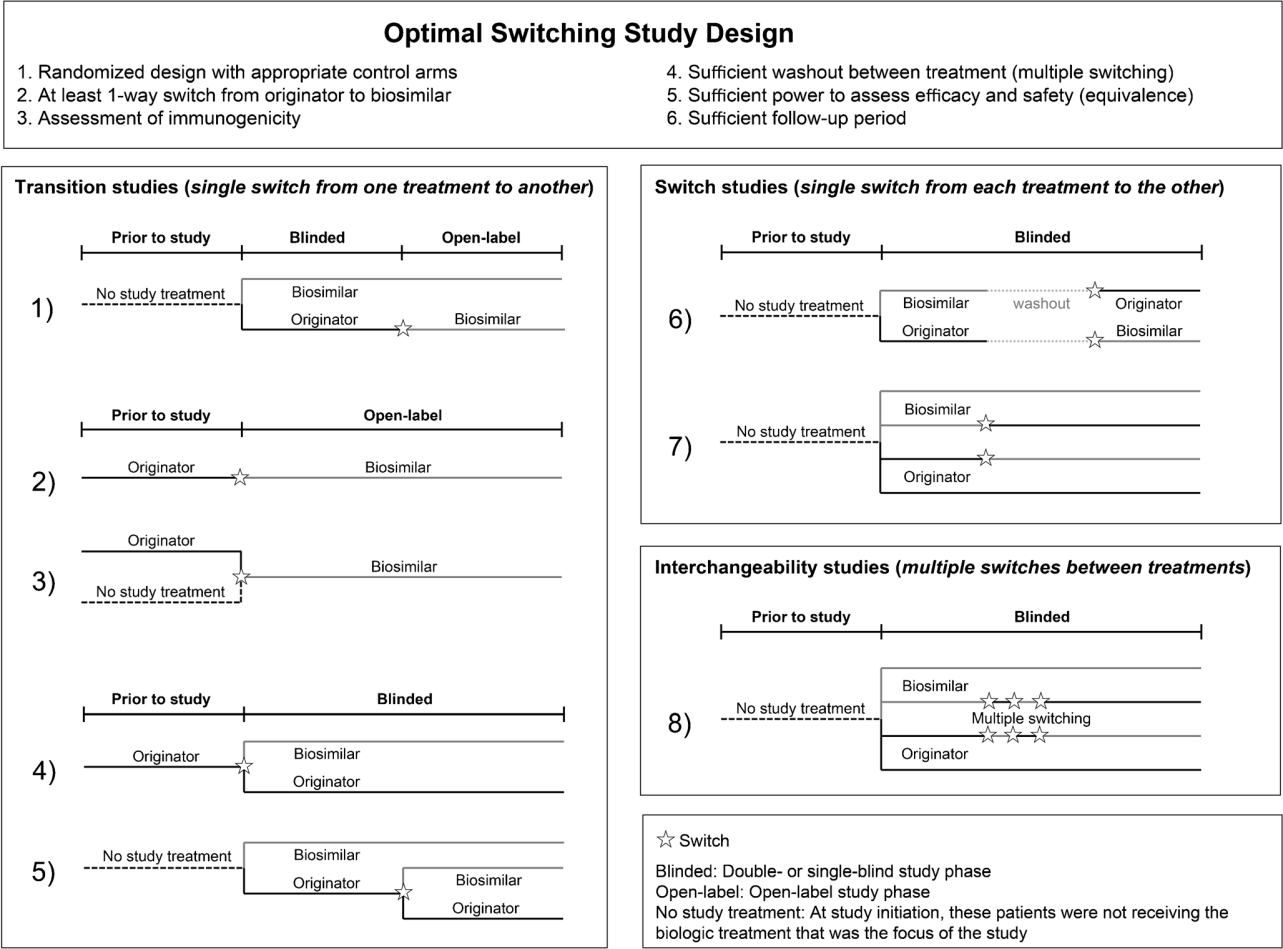
Elements of the optimal switching study and study designs employed by switching studies of reference biologics and biosimilars

Clinical and real-world studies conducted using scientifically sound methodology and that have an appropriate trial design provide the highest levels of evidence that a reference compound can be effectively switched with its biosimilar. Trial designs of biosimilar switching studies have been discussed previously [8–10], and six elements are considered necessary to fully demonstrate the safety of switching between reference biologic and biosimilar drugs [10–12] (Fig. 1). Studies can incorporate these elements in several different ways (Fig. 1) and can be transition studies (patients receiving treatment A switch to B, but not vice versa), switch studies (patients receiving treatment A switch once to B while those receiving B switch once to A), or interchangeability studies (patients switch treatments multiple times) [8, 13].

Several proposed biosimilars of the reference biologics infliximab (Remicade), etanercept (Enbrel), adalimumab (Humira), and rituximab (MabThera/Rituxan) are in development, and six compounds have been approved by either the European Medicines Agency (EMA) [14] or the FDA [15] (Table 1). The level of available evidence regarding switching varies greatly for these treatments. This review assesses the current body of switching data for these reference biologics and their respective biosimilars and proposed biosimilars.

**Table 1:** Approval status of proposed biosimilars of infliximab, etanercept, adalimumab, and rituximab

Biologic reference	Biologic copy	Regulatory body							
		EMA				FDA			
		Approval status	Date	Proprietary name(s)	Designation	Approval status	Date	Proprietary name(s)	Designation
Infliximab	CT-P13	*Approved* [14]	September 2013	Inflectra/Remsima	Biosimilar	*Approved* [15]	April 2016	Inflectra	Biosimilar
	SB2	*Approved* [14]	May 2016	Flixabi	Biosimilar	Accepted for regulatory review [16]	May 2016	-	Proposed
Etanercept	SB4	*Approved* [14]	January 2016	Benepali	Biosimilar	Not submitted for review	-	-	Proposed
	GP2015	MAA accepted for regulatory review [17]	December 2015	n/a	Proposed	*Approved* [15]	August 2016	Erelzi	Biosimilar
	CHS-0214	Planned MAA submission [18]	Q4 2016	-	Proposed	Not submitted for review	-	-	Proposed
	HD203	Not submitted for review	-	-	Proposed	Not submitted for review	-	-	Proposed
	LBEC0101	Not submitted for review	-	-	Proposed	Not submitted for review	-	-	Proposed
	TuNEX	Not submitted for review	-	-	Proposed	Not submitted for review	-	-	Proposed
Adalimumab	ABP 501	*Positive CHMP opinion* [19, 20]	January 2017	Amgevita/Solymbic	Proposed	*Approved* [15]	September 2016	Amjevita	Biosimilar
	SB5	MAA accepted for regulatory review [21]	July 2016	-	Proposed	Not submitted for review	-	-	Proposed
	M923	Not submitted for review	-	-	Proposed	Not submitted for review	-	-	Proposed
Rituximab	CT-P10	MAA submitted for review [22]	November 2015	-	Proposed	Not submitted for review	-	-	Proposed
	GP2013	MAA accepted for regulatory review [23]	May 2016	-	Proposed	Not submitted for review	-	-	Proposed

Abbreviations: *CHMP* Committee for Medicinal Products for Human Use, *EMA* European Medicines Agency, *FDA* United States Food and Drug Administration, *MAA* marketing authorization application, *Q4* fourth quarter

## Methodology

We conducted a search (31 October 2016) of PubMed and Web of Knowledge to identify studies where patients being treated with infliximab, etanercept, adalimumab, or rituximab for conditions in the areas of rheumatology, gastroenterology, and dermatology switched from the reference product to a biosimilar or proposed biosimilar. The International Clinical Trials Registry Platform was searched to identify unpublished clinical trials involving reference biologic → biosimilar switch(es). Selected congresses were hand-searched to identify abstracts not indexed at the time of the literature search. Search details are available in Online Resource Tables 1 and 2. The effect of switching on the three clinical areas of drug efficacy, safety, and immunogenicity was noted for each published study.

## Results

### Switching Between Reference and Biosimilar/Proposed Biosimilar Infliximab

Many clinical and observational studies involving a switch between reference infliximab and its biosimilars CT-P13 [24, 25•, 26, 27•, 28, 29, 30•, 31•, 32•, 33•, 34•, 35–64, 65•, 66•, 67–72] (EMA/FDA-approved), SB2 [73•, 74•] (EMA-approved), and NK [75] (Japanese Pharmaceuticals and Medical Devices Agency-approved) have been conducted or are ongoing (Table 2). Some studies report switching data but do not identify the biosimilar used [76, 77].

**Table 2:** Studies of reference and biosimilar infliximab, etanercept, adalimumab, and rituximab incorporating a switch protocol

Innovator	Biosimilar	Sponsor study name (trial registration)	Study design (study type^a^)	Indication(s)	Number of patients	Switches/patient	Follow-up post-switch	Status (predicted completion date)	Switching data published?
Infliximab	CT-P13	PLANETRA (NCT01217086)	OL extension to phase III DB RCT (transition, design 1)	RA	302	1	48 weeks	Completed	Yes [24, 25•]
Infliximab	CT-P13	PLANETAS (NCT01220518)	OL extension to phase I DB RCT (transition, design 1)	AS	174	1	48 weeks	Completed	Yes [26, 27•]
Infliximab	CT-P13	(JapicCTI-142419) Tanaka	OL extension to phase I/II RCT (transition, design 1)	RA	71	1	134 weeks	Completed	Yes [28]
Infliximab	CT-P13		Database analysis (transition, design 2)	RA	3018	1	Variable	Completed	Yes [29]
Infliximab	CT-P13	DANBIO	Registry analysis (transition, design 2)	RA, PsA, AxSpA	792	1	11 months	Completed	Yes [30•, 31•, 32•, 33•, 34•]
Infliximab	CT-P13		Single-center study (transition, design 2)	RA, AS, PsA, enteropathic arthritis	56	1	Variable	Completed	Yes [35]
Infliximab	CT-P13		Single-center study (transition, design 2)	AS, enteropathic arthritis, PsA, undifferentiated SpA	41	1	26 weeks	Completed	Yes [36–38]
Infliximab	CT-P13		Single-center study (transition, design 2)	RA, SpA, PsA, JRA, chronic reactive arthritis	39	1	Variable	Completed	Yes [39, 40]
Infliximab	CT-P13		Single-center study (transition, design 2)	Inflammatory arthritis	34	1	n/a	Completed	Yes [41]
Infliximab	CT-P13		Single-center study (transition, design 2)	RA, AS, PsA	30	1	n/a	Completed	Yes [42]
Infliximab	CT-P13	BIO-SWITCH (NTR5279)	Observational OL, phase IV (transition, design 4)	RA, SpA, PsA	192	1	52 weeks	Ongoing (April 2017)	Partly [43]
Infliximab	CT-P13		Single-center study (transition, design 2)	CD, UC	143	1	6 months	Completed	Yes [44]
Infliximab	CT-P13		Single-center MSP (transition, design 2)	IBD	134	1	16 weeks	Completed	Yes [45]
Infliximab	CT-P13		Retrospective OL (transition, design 3)	CD, UC	110	1	Variable	Completed	Yes [46, 47]
Infliximab	CT-P13		Observational cohort (transition, design 2)	CD, IBD, UC	83	1	16 weeks	Completed	Yes [48, 49]
Infliximab	CT-P13		Single-center study (transition, design 3)	CD	75	1	26 weeks	Completed	Yes [50]
Infliximab	CT-P13		Prospective (transition, design 2)	CD, UC	74	1	24 weeks	Completed	Yes [51, 52]
Infliximab	CT-P13		Single-center study (transition, design 3)	UC	40	1	26 weeks	Completed	Yes [53]
Infliximab	CT-P13		Observational OL (transition, design 2)	Pediatric CD and UC	39	1	32 weeks	Completed	Yes [54–57]
Infliximab	CT-P13		Single-center study (transition, design 3)	CD, UC	25	1	48	Completed	Yes [58]
Infliximab	CT-P13		Retrospective OL (transition, design 3)	CD, UC	17	1	n/a	Completed	Yes [59, 60]
Infliximab	CT-P13		Observational (transition, design 3)	CD, UC	397	n/a	Variable	Ongoing (unknown)	Partly [61, 62]
Infliximab	CT-P13		OL, phase 4 (transition, design 3)	CD, FCD, UC	173	1	30 weeks	Ongoing (unknown)	Partly [63]
Infliximab	CT-P13		Single-center study (transition, design 3)	PsO	35	1	Variable	Completed	Yes [64]
Infliximab	CT-P13	NOR-SWITCH (NCT02148640)	DB RCT, phase 4 (transition, design 4)	RA, SpA, PsA, UC, CD, PsO	481	1	52 weeks	Completed	Yes [65•, 66•]
Infliximab	CT-P13		Single-center study (transition, design 2)	PsA, AS, RA, CD, Behçet’s disease	23	1	n/a	Completed	Yes [67]
Infliximab	CT-P13	(JapicCTI-142,703)	OL extension to phase IV RCT (transition, design 1)	RA	n/a	1	n/a	Completed	No [68]
Infliximab	CT-P13	SIMILAR (2015–001954-14)	Phase IV DB RCT, OL extension planned (transition, design 4)	UC, CD	182	1	30 weeks	Ongoing (August 2016)	No [69]
Infliximab	CT-P13	(NCT02096861)	DB RCT, phase III (substitution, design 7)	CD	220	n/a	n/a	Ongoing (February 2017)	No [70]
Infliximab	CT-P13	CONNECT-IBD (NCT02539368)	Observational, phase IV (transition, design 2)	CD, UC	n/a	n/a	n/a	Ongoing (June 2019)	No [71]
Infliximab	CT-P13	SECURE (2014–004904-31)	OL (no comparator), phase IV (transition, design 2)	RA, UC, CD	n/a	1	16 weeks	Ongoing (March 2016)	No [72]
Infliximab	SB2	(NCT01936181)	DB RCT, phase III (transition, design 5)	RA	396	1	24 weeks	Completed	Yes [73•, 74•]
Infliximab	Infliximab NK		Single-center (transition, design 3)	UC, CD	20	1	22 weeks	Completed	Yes [75]
Infliximab	Unspecified		Retrospective OL (transition, design 2)	IBD	72	1	26 weeks	Completed	Yes [76]
Infliximab	Unspecified	(UMIN 000021492)	OL single-center study (transition, design 2)	RA	40	1	n/a	Ongoing (unknown)	No [77]
Etanercept	SB4	(NCT01895309)	OL extension of phase III RCT (transition, design 1)	RA	245	1	48 weeks	Completed	Yes [78•, 79•]
Etanercept	SB4	(NCT01865552)	SB RCT, phase III (substitution, design 6)	–	138	1	28 days	Completed	Yes [80, 81]
Etanercept	SB4	BIO-SPAN (NTR5901)	OL single-center study (transition, design 2)	Rheumatic disease	500	1	52 weeks	Ongoing (December 2017)	No [82]
Etanercept	GP2015	EGALITY (NCT01891864 2012-002011-26)	DB RCT, phase III (interchangeability, design 8)	PsO	531	3	40 weeks	Completed	Yes [83•]
Etanercept	GP2015		DB RCT, phase I (substitution, design 6)	–	54	1	28 days	Completed	Yes [84]
Etanercept	GP2015	EQUIRA (2012-002009-23)	DB RCT, phase III (transition, design 1)	RA	366	1	24 weeks	Ongoing (September 2017)	No [85]
Etanercept	HD203	(NCT01431404)	DB SD crossover, phase I (substitution, design 6)	–	42	1	21 days	Completed	No [86]
Etanercept	CHS-0214	RApsody (NCT02115750)	DB RCT, phase III (transition, design 1)	RA	647	1	n/a	Completed	No [87]
Etanercept	LBEC0101	(NCT01725620)	DB SD crossover, phase I (substitution, design 6)	–	48	1	27 days	Completed	No [88]
Etanercept	LBEC0101	(NCT01145950)	DB SD crossover, phase I (substitution, design 6)	–	36	1	22 days	Completed	No [89]
Etanercept	LBEC0101	(NCT02715908)	OL extension of phase III RCT (transition, design 1)	RA	165	1	48 weeks	Ongoing (March 2018)	No [90]
Etanercept	TuNEX	(ICTRP KCT0000118)	OL SD crossover, phase I (substitution, design 6)	–	23	1	21 days	Completed	No [91, 92]
Adalimumab	ABP 501	(NCT02114931)	OL extension of phase III RCT (transition, design 1)	RA	467	1	48 weeks	Completed	Yes [93•]
Adalimumab	ABP 501	(NCT01970488)	DB RCT, phase III (transition, design 5)	PsO	350	1	36 weeks	Completed	Yes [94, 95]
Adalimumab	SB5	(NCT02167139)	DB RCT, phase III (transition, design 5)	RA	273	1	28 weeks	Completed	Yes [96•, 97•]
Adalimumab	M923	(2015-001751-76)	DB RCT, phase III (transition, design 5)	PsO	516	Multiple	13 weeks	Ongoing (March 2017)	No [98]
Rituximab	CT-P10	(NCT01873443)	OL extension to phase I RCT (transition, design 1)	RA	87	1	56 weeks	Completed	Yes [99•]
Rituximab	GP2013	ASSIST-RT (NCT02514772)	DB RCT, phase III (transition, design 4)	RA	107	1	12 weeks	Ongoing (December 2016)	No [100]

^a^As illustrated in Fig. 1

Abbreviations: *AS* ankylosing spondylitis, *AxSpA* axial spondyloarthritis, *CD* Crohn’s disease, *DB* double-blind, *FCD* fistulizing active Crohn’s disease, *IBD* inflammatory bowel disease, *JRA* juvenile rheumatoid arthritis, *MSP* managed switching program, *n/a* information not available, *OL* open-label, *PsA* psoriatic arthritis, *PsO* psoriasis, *RA* rheumatoid arthritis, *RCT* randomized controlled trial, *SpA* spondyloarthritis, *UC* ulcerative colitis

Data have been published from 26 studies of reference infliximab → CT-P13 switching (23 completed, 3 ongoing) in various indications: rheumatic disease [24, 25•, 26, 27•, 28, 29, 30•, 31•, 32•, 33•, 34•, 35–43] (*n* = 11); inflammatory bowel disease [44–63] (*n* = 12); psoriasis (PsO) [64] (*n* = 1); and multiple disease areas [65•, 66•, 67] (*n* = 2). All these studies are transition studies (single switch from reference → biosimilar), although the precise designs used vary (Table 2]. Switching data are available in Online Resource Table 3).

Three of the 10 completed reference infliximab → CT-P13 switching studies in patients with rheumatic disease are open-label extensions (OLEs) of double-blind (DB) studies (study design 1, Fig. 1). In the PLANETRA [24, 25•], PLANETAS [26, 27•], and Tanaka et al. [28] studies, patients with rheumatoid arthritis (RA) or ankylosing spondylitis (AS) who completed a DB clinical trial of CT-P13 [101–103] enrolled in an OLE; patients who received reference infliximab in the DB stage switched to CT-P13. In all three studies, clinical measures of efficacy were similar in the switched and non-switched groups at study end. Efficacy was also similar pre- and post-switch in the switched groups. In the PLANETRA and Tanaka et al. studies, clinical measures of safety were comparable between non-switched and switched groups [24, 25•, 28]. In the PLANETAS study, the proportion of patients with ≥1 treatment-emergent adverse event (TEAE) was higher in the switched group (71.4%) than the non-switched group (48.9%) [26, 27•]. However, rates of TEAEs in both groups during both phases of the PLANETAS study were within the range reported historically in studies of reference infliximab in patients with AS [104–110] and were mild or moderate in severity [26, 27•]. In all studies, immunogenicity was comparable between the non-switched and switched groups at study end, and anti-drug antibody (ADAb) positivity did not increase in the OLE versus the DB stage [24, 25•, 26, 27•, 28].

The other seven completed studies of infliximab → CT-P13 switching in patients with rheumatic disease are either database/registry analyses [29, 30•, 31•, 32•, 33•, 34•] or small (30–56 patients) single-center studies [35–42] with no comparator arms (Fig. 1, study design 2). As with the clinical trials described above, efficacy and safety outcomes were usually similar pre- and post-switch, although one study reported a significant reduction in duration of morning stiffness following switch to CT-P13 [36]. The DANBIO registry study in patients with various rheumatic diseases found that 117/768 (15%) of patients discontinued treatment between switching and end of follow-up (median 336 days) for reasons including AEs (34 patients) and loss of efficacy (51) [33•]. Only two studies reported immunogenicity outcomes, with no change in ADAb levels reported before/after switching [31•, 32•, 36].

The final study in patients with rheumatic disease, which is still ongoing, is the BIO-SWITCH study. Interim data showed that patients with spondyloarthritis (SpA), but not RA or psoriatic arthritis (PsA), experienced significantly enhanced mean disease activity 6 months post-switch. The number of patients with ADAbs decreased from study baseline to end, but the significance of this was not discussed [43].

The 12 published studies of reference infliximab → CT-P13 switching in patients with inflammatory bowel disease were predominantly open-label (OL) observational studies utilizing study design 2 or 3 (Fig. 1) and were of various sizes (17–143 patients) and duration (Table 2) [44–63]. All studies except for one [45] reported efficacy outcomes, with no significant differences generally found between reference infliximab and CT-P13. In the PROSIT-BIO study, a significantly higher proportion of patients who switched from reference infliximab to CT-P13 (12%) experienced a loss of treatment response over 6 months, compared with patients receiving CT-P13 who were anti-TNF-α-naïve (1%) or who had previous exposure to a biologic (5%) [61, 62]. All 12 studies reported safety outcomes, and none reported any differences in safety profile between reference infliximab and CT-P13. Four studies reported immunogenicity data [44, 48, 49, 51, 52, 57]; two compared ADAb positivity pre- and post-switch, reporting no increase in immunogenicity from time of switch to study end [51, 52, 57].

One small single-center transition study was conducted in patients with PsO [64] (design 3, Fig. 1). Efficacy and safety outcomes were similar pre- and post-switch. Immunogenicity was not assessed.

Two studies were conducted across multiple indications [65•, 66•, 67]. The first was the NOR-SWITCH study; a large phase IV DB randomized controlled trial (RCT) (design 4, Fig. 1) in patients with various inflammatory conditions [Crohn’s disease (CD; *n* = 155, 32.2%), ulcerative colitis (UC; *n* = 93, 19.3%), SpA (*n* = 91, 18.9%), RA (*n* = 77, 16.0%), PsA (*n* = 30, 6.2%), and PsO (*n* = 35, 7.3%)]. Switching from reference infliximab to CT-P13 was not inferior to continued treatment with the reference product. Disease worsening (study primary endpoint), other measures of disease, and incidence of ADAbs were similar for patients receiving reference infliximab versus CT-P13 across all diseases [65•, 66•]. The second study was a small single-center transition study (design 2, Fig. 1) of patients in disease remission who switched treatments. Relapse occurred in seven (30%) patients after a mean of 1.7 months of CT-P13 treatment; these patients switched back to reference infliximab and improvements were seen in five (71%) of these patients. Immunogenicity was not assessed [67].

There are currently five unpublished studies (one completed, four ongoing) evaluating switching between reference infliximab and CT-P13. The completed study, in patients with RA (JapicCTI-142,703 [68]), is an OLE of a DB RCT [103] with patients receiving reference infliximab in the DB phase and CT-P13 in the OLE constituting the switch group (design 1, Fig. 1). The four ongoing unpublished studies of reference infliximab and CT-P13 are of various design [three transition studies [69, 71, 72] of designs 2 and 4, and one switch study [70] of design 7 (Fig. 1)]. Two studies are DB RCTs [69, 70] and two are OL observational studies [71, 72]. SIMILAR [69] will randomize patients in a blinded manner to either continue their current treatment or switch to CT-P13. Study NCT02096861 [70] will have four treatment arms: (1) reference infliximab throughout; (2) switch from reference infliximab → CT-P13; (3) CT-P13 throughout; (4) switch from CT-P13 → reference infliximab. CONNECT-IBD [71] will follow patients who have been prescribed reference infliximab or CT-P13 and document any switches and reasons for switching. SECURE [72] will switch all patients to CT-P13.

Overall, CT-P13 switching data suggest that it is well tolerated and effective in different patient populations and that reference → CTP-13 switching has no effect on treatment efficacy or safety. CT-P13 immunogenicity data have been reported in 12/26 studies and show no difference in ADAb incidence between reference infliximab and this biosimilar product (Online Resource Table 3). The question still remains as to why some patients who are stable on reference infliximab, sometimes for several years, discontinue treatment with the biosimilar after switching.

The biosimilars SB2 [73•, 74•] and infliximab NK [75] each have a single switching study. The SB2 study, in patients with RA, is a transition study [73•, 74•] (design 5, Fig. 1). Patients receiving reference infliximab for the first 54 weeks were re-randomized to receive either reference infliximab or SB2 for an additional 24 weeks. Patients receiving SB2 in the main study did not switch treatments. The efficacy, safety, and immunogenicity profiles were comparable between the three treatment groups. The infliximab NK study, in patients with CD or UC, is a transition study [75] (design 3, Fig. 1). In this single-center study, patients previously receiving reference infliximab switched to infliximab NK, and patients who were anti-TNF-α-naïve started treatment with infliximab NK. Remission was maintained in switched patients and achieved by 80–100% of NK-induced patients (depending on indication). No differences in safety were noted between groups. Immunogenicity outcomes were not reported.

As each product must be considered separately, the current body of data on these infliximab biosimilars (SB2 and NK, each with only a single switching study) requires further data and follow-up by pharmacovigilance, registry data and/or additional studies to demonstrate the safety of switching between these infliximab biosimilar products.

### Switching Between Reference and Biosimilar/Proposed Biosimilar Etanercept

The switching studies conducted on reference etanercept and its biosimilars, and proposed biosimilars cover six different products (Table 2). These are the EMA-approved biosimilar SB4, the FDA-approved biosimilar GP2015, and the Korean Ministry of Food and Drug Safety-approved biosimilar HD203 (not EMA/FDA-approved). Three other compounds, CHS-0214, LBEC0101, and TuNEX, are still under development (Table 1). Nine studies of these biosimilars and proposed biosimilars are completed (four with published switching data) and three are ongoing. Details regarding efficacy, safety, and immunogenicity of switching between reference etanercept and its various biosimilars and proposed biosimilars are available in Online Resource Table 3.

SB4 is the only EMA-approved biosimilar of etanercept. Three SB4 studies utilize a switching protocol and two have published switching data. The first is an OLE of a phase III DB study of etanercept and SB4 (transition study, design 1, Fig. 1) [78•, 79•]. The second is a phase I, randomized, single-blind crossover study (with washout) of the pharmacokinetics (PK) of etanercept and SB4 (switch study, design 6, Fig. 1) [80, 81]. In the phase III trial, patients with RA were randomized to receive etanercept or SB4 for an initial 52 weeks [111], after which patients receiving etanercept switched to SB4 for a further 48 weeks [78•, 79•]. Clinical measures of efficacy were similar for switched and non-switched patients at study end and were similar pre- and post-switch. Safety was similar for switched and non-switched patients at study end; the higher level of hepatobiliary disorders reported with SB4 compared with etanercept (17 vs. 0 adverse events (AEs) [112], all reported pre-switch [111]) was heterogeneous and thought to be due to chance rather than to true SB4 causality [113]. Switching from etanercept to SB4 did not result in any increase in immunogenicity [78•, 79•]. In the phase I trial, the PK of etanercept and SB4 was assessed in a three-part study. Part A involved a transition between European Union (EU)-sourced etanercept and SB4. Part B involved a transition between US-sourced etanercept and SB4. Part C involved a transition between EU- and US-sourced etanercept. In all parts, immunogenicity was assessed before and 28 days after the first dose, but not after the transition. PK parameters were similar for etanercept and SB4 in all parts, but as the study did not provide the data from pre- and post-switch evaluations separately, the effect of the switch on PK parameters was not assessed. There were no discontinuations post-switch [80, 81]. The final switching study of Enbrel and SB4 (BIO-SPAN [82]) is a switching study of etanercept and SB4 in patients with rheumatic disease. This ongoing study is an OL transition study (study design 2, Fig. 1) with all patients switching to SB4.

GP2015 is the only FDA-approved biosimilar of etanercept. Three GP2015 studies utilize a switching protocol and two have published switching data. The first (EGALITY [83•]) is a phase III DB RCT in patients with PsO, incorporating two non-switching arms and two switching arms involving multiple switches (interchangeability study, design 8, Fig. 1). Patients were randomized to treatment with either etanercept or GP2015 for 12 weeks and then re-randomized to either remain on their current treatment or to undergo repeated switching between treatments (three switches at 6-week intervals to week 30, and then maintain treatment to week 52). Repeated switching had no impact on efficacy, safety, or immunogenicity [83•]. The second GP2015 study is a phase I DB RCT PK study (switch study, design 6, Fig. 1) [84], with the PK of etanercept and GP2015 assessed with a single switch each way. PK parameters were similar for etanercept and GP2015 in all parts, but as the study did not provide the data from pre- and post-switch PK, safety, or immunogenicity evaluations separately, the effect of switching on these outcomes was not assessed [84].

The final switching study of etanercept and GP2015 (EQUIRA [85]) is an ongoing, phase III DB RCT with OLE (transition study, design 1, Fig. 1) in patients with RA, with patients randomized to treatment with etanercept in the DB phase switching to GP2015 in the OLE.

There is no evidence yet to support switching between reference etanercept and any of the proposed biosimilars HD203, CHS-0214, LBEC0101, and TuNEX, but six studies are either completed or ongoing. Four of these studies of etanercept proposed biosimilars are phase I single-dose crossover studies of HD203 [86], LBEC0101 [88, 89], and TuNEX [91, 92]. One study is a phase III DB RCT of CHS-0214 [87], and one is an OLE of a phase III DB RCT of LBEC0101 [90] (Table 2). No switching data are yet available from these studies.

Overall, data showing the safety of switching of reference etanercept to a biosimilar are only available for SB4 and GP2015, and for each data are only available from two studies (one phase I study and one phase III). The safety of transition and switching between reference etanercept and its proposed biosimilars requires follow-up by pharmacovigilance, registry data, and/or additional studies in order to provide sufficient long-term real-world data.

### Switching Between Reference and Biosimilar/Proposed Biosimilar Versions of Adalimumab and Rituximab

ABP 501 is the only EMA/FDA-approved biosimilar of adalimumab, and there are no EMA- or FDA-approved biosimilars of rituximab. Compared with infliximab and etanercept, there are very few adalimumab or rituximab reference → biosimilar/proposed biosimilar switching studies (Table 2). Details of the published studies are available in Online Resource Table 3.

There are two switching studies of adalimumab and its biosimilar ABP 501 (two transition studies, designs 1 [93•] and 5 [94, 95]; Fig. 1). One is a phase III DB RCT in patients with PsO [94, 95], while the other is an OLE to a phase III RCT in patients with RA [93•]. In the RA study, patients were randomized to DB treatment with either reference adalimumab or ABP 501 for 24 weeks. Patients randomized to treatment with reference adalimumab then switched to OL ABP 501, and patients receiving ABP 501 continued treatment. Post-switch, efficacy was reported as being maintained with no new safety findings. Long-term safety and efficacy results were reported to be similar between patients who switched from reference adalimumab and those who continued treatment with ABP 501; however, the necessary data to compare switched and non-switched patients are not available [93•]. In the PsO study, patients were first randomized to treatment with reference adalimumab or ABP 501 for 16 weeks; patients receiving reference adalimumab were then re-randomized to either continue treatment with reference adalimumab or switch to ABP 501. No efficacy or safety data were presented, but immunogenicity was not affected by switching treatments [94, 95].

The studies of the proposed adalimumab biosimilars SB5 and M923 are both phase III DB RCTs. The SB5 study is a transition study (design 5, Fig. 1) conducted in patients with RA [96•, 97•]. Patients were first randomized to treatment with reference adalimumab or SB5 for 24 weeks; patients receiving reference adalimumab were then re-randomized to either continue treatment with reference adalimumab or switch to SB5. Switching had no impact on efficacy, safety, or immunogenicity. The M923 study is an ongoing transition study (design 5, Fig. 1) in patients with PsO [98]. Patients will be randomized to treatment with reference adalimumab or M923; some patients receiving reference adalimumab will later switch to M923.

There are two switching studies of reference rituximab and its proposed biosimilars CT-P10 [99•] and GP2013 [100]. The CT-P10 study is a transition study (design 1, Fig. 1), an OLE [99•] of a phase I DB RCT [114] in patients with RA. Clinical measures of efficacy and safety were similar in the switched and non-switched groups. Efficacy pre- and post-switch could not be compared as different efficacy measures were reported for the DB [114] and OLE [99•] phases of the study. Immunogenicity was only assessed pre-switch. ASSIST-RT [100] is an ongoing switching study of GP2013 in which patients with RA already receiving reference rituximab will be randomized to either continue treatment or switch to GP2013.

Data regarding switching of reference adalimumab or rituximab and their biosimilars/proposed biosimilars are limited and the safety of switching has not yet been sufficiently demonstrated.

### Considerations Around Switching from Biologics to Biosimilars

Switching between reference biologics and biosimilar versions is a therapeutic transition based on prescriber decision. Medical switching should be performed by the prescriber for clinical reasons, such as optimizing efficacy or minimizing AEs. The potential for financial savings with biosimilars [115] makes it likely that switching between reference biologics and biosimilars will take place for non-medical reasons as no changes in clinical outcomes are expected after switching.

Any decision to switch biologic treatments should remain a clinical decision made by the treating physician on a case-by-case basis, with full patient awareness, and supported by scientific evidence. Many factors must be considered. Switching data is not transferable between different biologics or between different biosimilars of the same biologic. Differences in the incidence or type of AEs upon switching must also be considered; even large RCTs for biosimilars are not powered to show the significance of the difference when rare, unexpected AEs occur [12]. In addition, clinical trials have stringent inclusion and exclusion criteria that may not appropriately reflect the real-world patient seen by HCPs. For instance, trials conducted in patients who are naïve to biologic treatment are not reflective of patients who are stable on a reference biologic. Clinical trials also need to balance the need for using a sensitive population (one most able to demonstrate the looked-for phenomenon) versus an appropriate population (one with a condition for which the treatment is indicated) [116]. Switching studies in stable patients must be interpreted with caution as the definition of “stable” varies between studies and can refer to both the clinical status of the patient and the dose of the existing medication being taken. Furthermore, pediatric and elderly populations and those with comorbidities are often under-represented in clinical trials [117–119]. The ability of the patient to adapt to changes when switching to a biosimilar also needs to be considered, especially if patients require instruction regarding a new injection device. Patient status is therefore a key consideration when weighing the pros and cons of switching to a biosimilar. Even when comprehensive data from clinical studies are available, it may not be appropriate to switch all patients.

Careful pharmacovigilance and use of patient registries to document rare AEs are critical in order to gather clinical evidence of the benefits and risks of switching in all patients. There is a need for national databases capturing details of biologic switching so that a full picture of the safety of even relatively infrequent switching of biosimilar products can be obtained. Effective pharmacovigilance will be as important as it currently is for reference biologics.

### Recommendations Regarding Switching to Biosimilars

In order to safeguard patient safety, we propose the following recommendations regarding switching between reference and biosimilar treatments:

(1) The decision to switch should be based on scientifically sound (including real-world) data.

(2) Switching between reference biologic and biosimilar products, or between different biosimilar products, should remain a clinical decision to be made by the treating physician on an individual patient basis with patient awareness.

(3) Switching data from one biologic molecule should not be used to inform switching decisions between other biologic/biosimilar treatments.

(4) Automatic substitution at the pharmacy level should not take place as this decision would not be made by the treating physician.

(5) Patients should be closely followed post-switching to monitor for AEs; data should be made available for national registries that report into large pharmacovigilance databases.

(6) The decision to switch patients from a reference product to its biosimilar should be made on a case-by-case basis depending on the underlying disease, patient characteristics and comorbidities, type of reference drug, and patient willingness to switch.

## Conclusions

The introduction of biosimilar versions of established biologic drugs used in a range of inflammatory diseases provides an opportunity to greatly increase patient access to treatment. The safety of switching to these biosimilars has not yet been fully demonstrated in terms of their long-term efficacy, safety, and immunogenicity. Thus, data from pharmacovigilance programs are needed in order to adequately inform clinical decision-making in relation to switching between these compounds. However, as the information gap is filled (particularly with data derived from appropriately designed switching clinical trials and real-world experience), we believe treatment practice will adapt.

## Electronic Supplementary Material

ESM 1 (DOCX 13 kb)

ESM 2 (DOCX 12 kb)

ESM 3 (DOCX 80 kb)

## References

[1] European Medicines Agency. Guideline on similar biological medicinal products. 2014. http://www.ema.europa.eu/docs/en_GB/document_library/Scientific_guideline/2014/10/WC500176768.pdf. Accessed 31 Mar 2016.

[2] United States Food and Drug Administration. Scientific considerations in demonstrating biosimilarity to a reference product. Guidance for industry. 2015. http://www.fda.gov/downloads/drugs/guidancecomplianceregulatoryinformation/guidances/UCM291128.pdf. Accessed 31 Mar 2016.

[3] World Health Organization. Guidelines on evaluation of similar biotherapeutic products (SBPs). 2010. http://www.who.int/biologicals/areas/biological_therapeutics/BIOTHERAPEUTICS_FOR_WEB_22APRIL2010.pdf. Accessed 31 Mar 2016.

[4] Scheinberg MA, Kay J. The advent of biosimilar therapies in rheumatology–“O Brave New World”. Nat Rev Rheumatol. 2012;8:430–6. 10.1038/nrrheum.2012.84 22664834

[5] Mysler E, Pineda C, Horiuchi T, Singh E, Mahgoub E, Coindreau J (2016). Clinical and regulatory perspectives on biosimilar therapies and intended copies of biologics in rheumatology. Rheumatol Int.

[6] United States Food and Drug Administration. Guidance for industry on biosimilars: Q & As regarding implementation of the BPCI Act of 2009: Questions and answers part I. 2016. http://www.fda.gov/Drugs/GuidanceComplianceRegulatoryInformation/Guidances/ucm259809.htm. Accessed 07 Oct 2016.

[7] United States Food and Drug Administration. Considerations in demonstrating interchangeability with a reference product: guidance for industry (draft guidance). 2017. https://www.fda.gov/downloads/Drugs/GuidanceComplianceRegulatoryInformation/Guidances/UCM537135.pdf. Accessed 28 Feb 2017.

[8] Dorner T, Kay J (2015). Biosimilars in rheumatology: current perspectives and lessons learnt. Nat Rev Rheumatol.

[9] Ebbers HC, Chamberlain P (2014). Interchangeability. An insurmountable fifth hurdle?. GaBI J.

[10] Endrenyi L, Chang C, Chow SC, Tothfalusi L (2013). On the interchangeability of biologic drug products. Stat Med.

[11] Alten R, Cronstein BN (2015). Clinical trial development for biosimilars. Semin Arthritis Rheum.

[12] Dorner T, Strand V, Cornes P, Goncalves J, Gulacsi L, Kay J, et al. The changing landscape of biosimilars in rheumatology. Ann Rheum Dis. 2016;75:974–82. doi:10.1136/annrheumdis-2016-209166. 10.1136/annrheumdis-2016-209166 PMC4893105 26964144

[13] Faccin F, Tebbey P, Alexander E, Wang X, Cui L, Albuquerque T. The design of clinical trials to support the switching and alternation of biosimilars. Expert Opin Biol Ther. 2016;16:1445–53. 10.1080/14712598.2017.1238454 27666115

[14] European Medicines Agency. European public assessment reports (biosimilars). http://www.ema.europa.eu/ema/index.jsp?curl=pages%2Fmedicines%2Flanding%2Fepar_search.jsp&mid=WC0b01ac058001d124&searchTab=searchByAuthType&alreadyLoaded=true&isNewQuery=true&status=Authorised&status=Withdrawn&status=Suspended&status=Refused&keyword=Enter+keywords&searchType=name&taxonomyPath=&treeNumber=&searchGenericType=biosimilars&genericsKeywordSearch=Submit. Accessed 31 Oct 2016.

[15] United States Food and Drug Administration. Center for drug evaluation and research list of licensed biological products. 2016. http://www.fda.gov/downloads/Drugs/DevelopmentApprovalProcess/HowDrugsareDevelopedandApproved/ApprovalApplications/TherapeuticBiologicApplications/Biosimilars/UCM439049.pdf. Accessed 31 Oct 2016.

[16] Samsung Bioepis. Samsung Bioepis’ biologics license application for SB2 infliximab biosimilar accepted by FDA. 2016. http://www.businesswire.com/news/home/20160523006086/en/Samsung-Bioepis-Biologics-License-Application-SB2-Infliximab. Accessed 31 Oct 2016.

[17] Novartis. Sandoz advances its biosimilars program with European Medicines Agency (EMA) acceptance of regulatory submission for biosimilar etanercept. 2015. https://www.novartis.com/news/media-releases/sandoz-advances-its-biosimilars-program-european-medicines-agency-ema-acceptance. Accessed 31 Oct 2016.

[18] Globe Newswire. Coherus BioSciences regains development and commercial rights to CHS-0214 from Shire. 2016. https://globenewswire.com/news-release/2016/09/27/874893/0/en/Coherus-BioSciences-Regains-Development-and-Commercial-Rights-to-CHS-0214-From-Shire.html. Accessed 31 Oct 2016.

[19] European Medicines Agency. Human medicines: pending EC decisions: Amgevita. http://www.ema.europa.eu/ema/index.jsp?curl=pages/medicines/human/medicines/004212/smops/Positive/human_smop_001091.jsp&mid=WC0b01ac058001d127&source=homeMedSearch&category=human. Accessed 28 Feb 2017.

[20] European Medicines Agency. Human medicines: pending EC decisions: Solymbic. http://www.ema.europa.eu/ema/index.jsp?curl=pages/medicines/human/medicines/004373/smops/Positive/human_smop_001092.jsp&mid=WC0b01ac058001d127&source=homeMedSearch&category=human. Accessed 28 Feb 2017.

[21] Biogen. SB5, an adalimumab biosimilar referencing Humira®, accepted for review by European Medicines Agency. 2016. http://media.biogen.com/press-release/biosimilars/sb5-adalimumab-biosimilar-referencing-humira-accepted-review-european-medi. Accessed 31 Oct 2016.

[22] Generics and Biosimilars Initiative. Celltrion submits rituximab biosimilar application to EMA. 2015. http://www.gabionline.net/Biosimilars/News/Celltrion-submits-rituximab-biosimilar-application-to-EMA. Accessed 31 Oct 2016. doi:10.3402/ijch.v74.27284.

[23] Novartis. New data demonstrates Sandoz’ etanercept and rituximab biosimilar candidates bioequivalent to originator products. 2016. https://www.novartis.com/news/media-releases/new-data-demonstrates-sandoz-etanercept-and-rituximab-biosimilar-candidates. Accessed 31 Oct 2016.

[24] Yoo DH, Prodanovic N, Jaworski J, Miranda P, Ramiterre E, Lanzon A, et al. Efficacy and safety of CT-P13 (infliximab biosimilar) over two years in patients with rheumatoid arthritis: comparison between continued CT-P13 and switching from infliximab to CT-P13. American College of Rheumatology 2013; San Diego, California, USA: 2013.

[25] Yoo DH, Prodanovic N, Jaworski J, Miranda P, Ramiterre E, Lanzon A (2017). Efficacy and safety of CT-P13 (biosimilar infliximab) in patients with rheumatoid arthritis: comparison between switching from reference infliximab to CT-P13 and continuing CT-P13 in the PLANETRA extension study. Ann Rheum Dis.

[26] Park W, Miranda P, Brzosko M, Wiland P, Gutierrez-Ureña S, Mikazane H, et al. Efficacy and safety of CT-P13 (infliximab biosimilar) over two years in patients with ankylosing spondylitis: comparison between continuing with CT-P13 and switching from infliximab to CT-P13. American College of Rheumatology 2013; San Diego, California, USA: 2013.

[27] Park W, Yoo DH, Miranda P, Brzosko M, Wiland P, Gutierrez-Ureña S (2017). Efficacy and safety of switching from reference infliximab to CT-P13 compared with maintenance of CT-P13 in ankylosing spondylitis: 102-week data from the PLANETAS extension study. Ann Rheum Dis.

[28] Tanaka Y, Yamanaka H, Takeuchi T, Inoue M, Saito K, Saeki Y, et al. Safety and efficacy of CT-P13 in Japanese patients with rheumatoid arthritis in an extension phase or after switching from infliximab. Mod Rheumatol. 2017;27:237-45. 10.1080/14397595.2016.1206244 27586879

[29] Yazici Y, Xie L, Ogbomo A, Parenti D, Goyal K, Teeple A, et al. A descriptive analysis of real-world treatment patterns in a Turkish rheumatology population that continued innovator infliximab (Remicade) therapy or switched to biosimilar infliximab. American College of Rheumatology; 2016 Washington DC, USA: Arthritis Rheumatol. 2016;68 (suppl10). http://acrabstracts.org/abstract/a-descriptive-analysis-of-real-world-treatment-patterns-in-a-turkish-rheumatology-population-that-continued-innovator-infliximab-remicade-therapy-or-switched-to-biosimilar-infliximab/ Accessed 17 May 2017.

[30] Glintborg B, Sørensen IJ, Jensen DV, Krogh NS, Loft AG, Colic A (2016). Three months’ clinical outcomes from a nationwide non-medical switch from originator to biosimilar infliximab in patients with inflammatory arthritis. Results from the DANBIO registry.

[31] • Glintborg B, Kringelbach T, Høgdall E, Sørensen IJ, Jensen DV, Loft AG, et al. Non-medical switch from originator to biosimilar infliximab in patients with inflammatory arthritis: impact on s-infliximab and antidrug antibodies. Results from the Danish Rheumatological Biobank and the DANBIO registry. Scandinavian Congress of Rheumatology; Reykjavik: 2016. **The DANBIO registry analysis is the largest real-world study of the effects of switching from infliximab to CT-P13 in patients with rheumatic disease to date. This publication provides important early immunogenicity data on the effect of switching.**

[32] • Glintborg B, Kringelbach T, Høgdall E, Sørensen IJ, Jensen DV, Loft AG, et al. Non-medical switch from originator to biosimilar infliximab among patients with inflammatory rheumatic disease–impact on s-infliximab and anti-drug antibodies. Results from the National Danish Rheumatologic Biobank and the DANBIO registry. European League Against Rheumatism 2016; London, UK; Ann Rheum Dis: 2016. **The DANBIO registry analysis is the largest real-world study of the effects of switching from infliximab to CT-P13 in patients with rheumatic disease to date. This publication provides important early immunogenicity data on the effects of switching.**

[33] • Glintborg B, Sørensen IJ, Jensen DV, Krogh NS, Loft AG, Espesen J, et al. A nationwide non-medical switch from originator to biosimilar infliximab in patients with inflammatory arthritis. Eleven months’ clinical outcomes from the Danbio Registry. American College of Rheumatology 2016; Washington DC, USA: 2016. **The DANBIO registry analysis is the largest real-world study of the effects of switching from infliximab to CT-P13 in patients with rheumatic disease to date. This publication provides important longer-term clinical data on the efficacy and safety of switching.**

[34] • Glintborg B, Kringelbach TM, Høgdall E, Sørensen IJ, Jensen DV, Loft AG, et al. Non-medical switch from originator to biosimilar infliximab in patients with inflammatory arthritis—impact on s-infliximab and antidrug-antibodies. Results from the Danish Rheumatologic Biobank and the Danbio Registry. American College of Rheumatology 2016; Washington DC, USA: 2016. **The DANBIO registry analysis is the largest real-world study of the effects of switching from infliximab to CT-P13 in patients with rheumatic disease to date. This publication provides important longer-term immunogenicity data on the effects of switching.**

[35] Holroyd C, Parker L, Bennett S, Zarroug J, Underhill C, Davidson B, et al. Switching to biosimilar infliximab: real-world data from the Southampton biologic therapies review service. British Society for Rheumatology 2016 Annual Meeting; Glasgow, UK; Rheumatology (Oxford): 2016. doi:10.1093/rheumatology/kew121.003.

[36] Benucci M, Gobbi FL, Bandinelli F, Damiani A, Infantino M, Grossi V, et al. Safety, efficacy and immunogenicity of switching from innovator to biosimilar infliximab in patients with spondyloarthritis: a 6-month real-life observational study. Immunol Res. 2016:1–4. doi:10.1007/s12026-016-8843-5. 10.1007/s12026-016-8843-5 27449503

[37] Batticciotto A, Parisi S, Gobbi FL, Antivalle M, Benucci M, Fusaro E, et al. Safety and efficacy of switching from innovator to biosimilar infliximab in patients affected by spondyloarthritis. A 6-month observational study. European League Against Rheumatism 2016; London, UK; Ann Rheum Dis: 2016. doi:10.1136/annrheumdis-2016-eular.5115.

[38] Batticciotto A, Antivalle M, Gobbi FL, Parisi S, Talotta R, Varisco V, et al. Safety and efficacy of switching from originator to CT-P13 infliximab biosimilar in patients affected by spondyloarthritis. A 6-month observational study. American College of Rheumatology 2016; Washington DC, USA: Arthritis Rheumatol. 2016.

[39] Sokka T, Kautiainen H. Clinical experience with infliximab biosimilar—switch from Remicade. European League Against Rheumatism 2015; Rome, Italy; Ann Rheum Dis: 2015. doi:10.1136/annrheumdis-2015-eular.2151.

[40] Nikiphorou E, Kautiainen H, Hannonen P, Asikainen J, Kokko A, Rannio T (2015). Clinical effectiveness of CT-P13 (infliximab biosimilar) used as a switch from Remicade (infliximab) in patients with established rheumatic disease. Report of clinical experience based on prospective observational data. Expert Opin Biol Ther.

[41] Abdalla A, Byrne NE, Conway R, Walsh T, Mannion G, Hanly M, et al. Long term safety and efficacy of biosimilar infliximab among patients with inflammatory arthritis switched from reference product. European League Against Rheumatism 2016; London, UK; Ann Rheum Dis: 2016. doi:10.2147/OARRR.S124975. 10.2147/OARRR.S124975 PMC5349501 28331376

[42] Malaiya R, McKee Z, Kiely P. Infliximab biosimilars—switching Remicade to Remsima in routine care: patient acceptability and early outcome data. British Society for Rheumatology 2016 Annual Meeting; Glasgow, UK; Rheumatology (Oxford): 2016.

[43] Tweehuysen L, van den Bemt BJF, van Ingen IL, de Jong AJL, van der Laan WH, van den Hoogen FHJ, et al. Clinical and immunogenicity outcomes after switching treatment from innovator infliximab to biosimilar infliximab in rheumatic diseases in daily clinical practice. American College of Rheumatology 2016; Washington DC, USA: 2016.

[44] Buer LC, Moum BA, Cvancarova M, Warren DJ, Medhus AW, Hoivik ML. Switching from Remicade(R) to Remsima(R) is safe and feasible: a prospective, open-label study. J Crohns Colitis. 2017;11:297–304. 10.1093/ecco-jcc/jjw166 27660339

[45] Bettey M, Downey L, Underhill C, Callaghan J, Rush M, Ahmed I, et al. Outcomes of a managed switching programme changing IBD patients established on originator infliximab to biosimilar infliximab. 11th Congress of the European Crohn’s and Colitis Organisation; Amsterdam, The Netherlands: 2016.

[46] Jung YS, Park DI, Kim YH, Lee JH, Seo PJ, Cheon JH (2015). Efficacy and safety of CT-P13, a biosimilar of infliximab, in patients with inflammatory bowel disease: a retrospective multicenter study. J Gastroenterol Hepatol.

[47] Jung YS, Park DI, Kim YH, Seo PJ, Kim JW, Kang HW. Efficacy and safety of infliximab’s biosimilar (REMSIMA) for IBD. 10th Congress of the European Crohn’s and Colitis Organisation; Barcelona, Spain; J Crohns Colitis: 2015.

[48] Smits L, Derikx L, Drenth J, de Jong D, van Esch A, Hoentjen F. Elective switching from Remicade® to biosimilar CT-P13 in inflammatory bowel disease patients: a prospective observational cohort study. 11th Congress of the European Crohn’s and Colitis Organisation; Amsterdam, The Netherlands: 2016.

[49] Smits LJT, Derikx LAAP, de Jong DJ, Boshuizen RS, van Esch AAJ, Drenth JPH (2016). Clinical outcomes following a switch from Remicade® to the biosimilar CT-P13 in inflammatory bowel disease patients: a prospective observational cohort study. J Crohns Colitis.

[50] Guerra Veloz MF, Argüelles Arias F, Perea Amarillo R, Castro Laria L, Maldonado Pérez MB, Benítez Roldán A, et al. Safety and efficacy of infliximab biosimilar (Remsima©) in Crohn’s disease patients in clinical practice: results after 6 months of treatment. 11th Congress of the European Crohn’s and Colitis Organisation; Amsterdam, The Netherlands: 2016a.

[51] Kolar M, Duricová D, Brotlik M, Hruba V, Machkova N, Mitrova K, et al. Switching of patients with inflammatory bowel disease from original infliximab (Remicade®) to biosimilar infliximab (Remsima™) is effective and safe. 11th Congress of the European Crohn’s and Colitis Organisation; Amsterdam, The Netherlands: 2016.

[52] Kolar M, Duricová D, Bortlik M, Hruba V, Machkova N, Mitrova K, et al. Switching of patients with inflammatory bowel disease from original infliximab (Remicade®) to biosimilar infliximab (Remsima™) is effective and safe—one-year follow-up. United European Gastroenterology Week; Vienna, Austria; United European Gastroenterol J: 2016.

[53] Guerra Veloz MF, Argüelles Arias F, Perea Amarillo R, Castro Laria L, Maldonado Pérez MB, Benítez Roldán A, et al. Safety and efficacy of infliximab biosimilar (Remsima©) in ulcerative colitis disease patients in clinical practice: results after 6-months treatment. 11th Congress of the European Crohn’s and Colitis Organisation; Amsterdam, The Netherlands: 2016b.

[54] Jarzebicka D, Banaszkiewicz A, Plocek A, Sieczkowska J, Gawronska A, Toporowska-Kowalska E, et al. Preliminary assessment of efficacy and safety of switching between originator and biosimilar infliximab in paediatric Crohn disease patients. 10th Congress of the European Crohn’s and Colitis Organisation; Barcelona, Spain: 2015.

[55] Kierkus J. Preliminary assessment of efficacy and safety of switching between originator and biosimilar infliximab in paediatric Crohn disease patients. 46th Annual Digestive Disease Week; Washington DC, USA; Gastroenterology: 2015. doi:10.1016/S0016-5085(15)32670-6.

[56] Sieczkowska J, Jarzebicka D, Banaszkiewicz A, Plocek A, Gawronska A, Toporowska-Kowalska E (2016). Switching between infliximab originator and biosimilar in paediatric patients with inflammatory bowel disease. Prelim Observ J Crohns Colitis.

[57] Sieczkowska J, Jarzebicka D, Oracz G, Meglicka M, Dadalski M, Kierkus J. Immunogenicity after switching from reference infliximab to biosimilar in children with Crohn’s disease. 11th Congress of the European Crohn’s and Colitis Organisation; Amsterdam, The Netherlands: 2016. doi:10.1093/ecco-jcc/jjw019.736.

[58] Hlavaty T, Krajcovicova A, Sturdik I, Letkovsky J, Koller T, Toth J, et al. Biosimilar infliximab CT-P13 treatment in patients with inflammatory bowel diseases: a 1-year, single-centre retrospective study. 11th Congress of the European Crohn’s and Colitis Organisation; Amsterdam, The Netherlands: 2016.

[59] Kang HW, Lim YJ, Kim JH, Kang YS. An experience of anti-TNF biosimilar, CT-P13 use: clinical efficacy, safety and interchangeability in inflammatory bowel disease; a pilot study. 9th Congress of the European Crohn’s and Colitis Organisation; Copenhagen, Denmark; J Crohns Colitis: 2014.

[60] Kang YS, Moon HH, Lee SE, Lim YJ, Kang HW (2015). Clinical experience of the use of CT-P13, a biosimilar to infliximab in patients with inflammatory bowel disease: a case series. Dig Dis Sci.

[61] Armuzzi A. What future for biosimilars in inflammatory bowel diseases? The Italian Group for the study of Inflammatory Bowel Disease VII National Congress; Palermo, Italy: 2015.

[62] Fiorino G, Manetti N, Variola A, Bossa F, Rizzuto G, Guidi L, et al. Prospective observational study on inflammatory bowel disease patients treated with infliximab biosimilars: preliminary results of the PROSIT-BIO cohort of the IG-IBD. 11th Congress of the European Crohn’s and Colitis Organisation; Amsterdam, The Netherlands: 2016. doi:10.1097/MIB.0000000000000995.

[63] Park SH, Kim YH, Lee JH, Kwon HJ, Lee SH, Park DI (2015). Post-marketing study of biosimilar infliximab (CT-P13) to evaluate its safety and efficacy in Korea. Exp Rev Gastroenterol Hepatol.

[64] Dapavo PMD, Vujic IMD, Fierro MTMD, Quaglino PMD, Sanlorenzo MMD (2016). The infliximab biosimilar in the treatment of moderate to severe plaque psoriasis. J Am Acad Dermatol.

[65] • Jørgensen K, Olsen I, Goll G, Lorentzen M, Bolstad N, Haavardsholm E, et al. Biosimilar infliximab (CT-P13) is not inferior to originator Infliximab: results from the 52-week randomized NOR-SWITCH trial. United European Gastroenterology Week; Vienna, Austria; United European Gastroenterol J: 2016. **The NOR-SWITCH study is the largest clinical trial of switching from infliximab to CT-P13 and includes patients from multiple indications. This study provides important efficacy, safety and immunogenicity data on switching.**

[66] • Goll GL, Olsen IC, Jorgensen KK, Lorentzen M, Bolstad N, Haavardsholm EA, et al. Biosimilar infliximab (CT-P13) is not inferior to originator infliximab: results from a 52-week randomized switch trial in Norway. American College of Rheumatology 2016; Washington DC, USA; Arthritis Rheumatol: 2016. **The NOR-SWITCH study is the largest clinical trial of switching from infliximab to CT-P13 and includes patients from multiple indications. This study provides important efficacy, safety and immunogenicity data on switching.**

[67] Gentileschi S, Barreca C, Bellisai F, Biasi G, Brizi MG, De Stefano R (2015). Switch from infliximab to infliximab biosimilar: efficacy and safety in a cohort of patients with different rheumatic diseases: response to: Nikiphorou E, Kautiainen H, Hannonen P, et al. Clinical effectiveness of CT-P13 (infliximab biosimilar) used as a switch from Remicade (infliximab) in patients with established rheumatic disease. Report of clinical experience based on prospective observational data. Expert Opin Biol Ther.

[68] JAPIC Clinical Trials Information. Extension study following the phase I/II clinical study of CT-P13 in the patients with rheumatoid arthritis (JapicCTI-142703). http://www.clinicaltrials.jp/user/cteSearch_e.jsp. Accessed 24 Mar 2016.

[69] EU Clinical Trials Register. SIMILAR Trial: Santeon InflixMab biosimILAr Research. A randomized, controlled, double blind, phase 4 noninferiority trial to assess efficacy of Infliximab-biosimilar (Inflectra) compared to Infliximab-innovator (Remicade) in patients with inflammatory bowel disease in remission. http://www.clinicaltrialsregister.eu/ctr-search/trial/2009-013129-41/NL. Accessed 22 Mar 2016.

[70] ClinicalTrials.gov. Demonstrate noninferiority in efficacy and to assess safety of CT-P13 in patients with active Crohn’s disease. http://clinicaltrials.gov/ct2/show/NCT02096861. Accessed 28 Apr 2016.

[71] ClinicalTrials.gov. Post-marketing use of inflectra (infliximab) for standard of care treatment of inflammatory bowel disease (CONNECT-IBD). http://clinicaltrials.gov/ct2/show/NCT02539368. Accessed 23 Mar 2016.

[72] EU Clinical Trials Register. An open-label, multicentre, phase IV study to investigate the infliximab serum concentration of Remsima™ (infliximab biosimilar) after switching from Remicade (infliximab) in subjects with Crohn’s Disease (CD), Ulcerative Colitis (UC) or Rheumatoid Arthritis (RA) in stable remission. http://www.clinicaltrialsregister.eu/ctr-search/trial/2014-004904-31/NL. Accessed 22 Mar 2016.

[73] • Smolen J, Choe JY, Prodanovic N, Niebrzdowski J, Staykov I, Dokoupilova E, et al. Comparable safety and immunogenicity and sustained efficacy after transition to SB2 (an infliximab biosimilar) vs ongoing infliximab reference product in patients with rheumatoid arthritis: results of phase III transition study. European League Against Rheumatism 2016; London, UK; Ann Rheum Dis: 2016. doi:10.1136/annrheumdis-2016-eular.3122. **Only published study to date of switching from infliximab to SB2. This study provides important efficacy, safety and immunogenicity data on switching**.

[74] • Smolen JS, Choe JY, Prodanovic N, Niebrzydowski J, Staykov I, Dokoupilova E, et al. Comparable safety and immunogenicity and sustained efficacy after transition to SB2 (an infliximab biosimilar) vs ongoing reference infliximab (Remicade®) in patients with rheumatoid arthritis: results of phase III transition study. American College of Rheumatology 2016; Washington DC, USA: 2016. **Only published study to date of switching from infliximab to SB2. This study provides important efficacy, safety and immunogenicity data on switching.**

[75] Hamanaka S, Nakagawa T, Koseki H, Sakurai T, Taida T, Okimoto K, et al. Infliximab biosimilar in the treatment of inflammatory bowel disease: a Japanese single-cohort observational study. 11th Congress of the European Crohn’s and Colitis Organisation; Amsterdam, The Netherlands: 2016.

[76] Díaz Hernández L, Rodríguez González GE, Vela González M, Tardillo Marín CA, Rodríguez Díaz CY, Arranz Hernández L et al. Efficacy and safety of switching between originator and biosimilar infliximab in patients with inflammatory bowel disease in practical clinic: results to 6 months. 11th Congress of the European Crohn’s and Colitis Organisation; Amsterdam, The Netherlands: 2016. doi:10.1093/ecco-jcc/jjw019.568.

[77] UMIN-CTR Clinical Trial. To investigate the safety of switch from infliximab biosimilar 1 in rheumatoid arthritis patients. https://upload.umin.ac.jp/cgi-open-bin/ctr_e/ctr_view.cgi?recptno=R000024788. Accessed 09 Sept 2016.

[78] • Emery P, Vencovský J, Sylwestrzak A, Leszczynski P, Porawska W, Stasiuk B, et al. Longterm safety and efficacy of SB4 (etanercept biosimilar) in patients with rheumatoid arthritis: comparison between continuing SB4 and switching from etanercept reference product to SB4. European League Against Rheumatism 2016; London, UK; Ann Rheum Dis: 2016. **Only published study to date of switching from etanercept to SB4 in patients with RA. This study provides important efficacy, safety and immunogenicity data on switching.**

[79] • Emery P, Vencovský J, Sylwestrzak A, Leszczynski P, Porawska W, Stasiuk B, et al. Additional efficacy results of SB4 (etanercept biosimilar) up to week 100: comparison between continuing SB4 and switching from reference etanercept (Enbrel®) to SB4. American College of Rheumatology 2016; Washington DC, USA: 2016. Ann Rheum Dis: 2016;75(Suppl2):236. **Only published study to date of switching from etanercept to SB4 in patients with RA. This study provides important efficacy, safety and immunogenicity data on switching**.

[80] Lee Y, Shin D, Kim Y, Kang J, Gauliard A, Fuhr R. A randomised Phase l pharmacokinetic study comparing SB4 and etanercept reference product (Enbrel(R)) in healthy subjects. Br J Clin Pharmacol. 2016;82:64–73. doi:10.1111/bcp.12929. 10.1111/bcp.12929 PMC4917797 26972584

[81] Lee YJ, Shin D, Kim Y, Kang JW, Fuhr R, Gauliard A. A phase I pharmacokinetic study comparing SB4, an etanercept biosimilar, and etanercept reference product (Enbrel®) in healthy male subjects. European League Against Rheumatism 2015; Rome, Italy; Ann Rheum Dis: 2015.

[82] Nederlands Trial Register. Switching treatment from innovator etanercept (Enbrel) to etanercept biosimilar (Benepali) in patients with a rheumatic disease in daily clinical care. http://www.trialregister.nl/trialreg/admin/rctview.asp?TC=5901. Accessed 19 Sept 2016.

[83] • Griffiths CE, Thaci D, Gerdes S, Arenberger P, Pulka G, Kingo K, et al. The EGALITY study: a confirmatory, randomised, double-blind study comparing the efficacy, safety and immunogenicity of GP2015, a proposed etanercept biosimilar, versus the originator product in patients with moderate to severe chronic plaque-type psoriasis. Br J Dermatol. 2017;176:928–38. **Only published study to date of switching from etanercept to GP2015 in patients. Interchangeability design with 2 control treatment arms allows effect of multiple switching to be assessed. This study also provides important efficacy, safety and immunogenicity data on switching.**

[84] Afonso M, Sanguino Heinrich S, Poetzl J, Woehling H. Pharmacokinetics and safety of GP2015, a proposed etanercept biosimilar, and etanercept originator product in healthy male subjects: a randomised twoway crossover study. European League Against Rheumatism 2016; London, UK; Ann Rheum Dis: 2016. doi:10.1136/annrheumdis-2016-eular.1422.

[85] EU Clinical Trials Register. A randomized, double-blind, parallel-group phase III study to demonstrate equivalent efficacy and to compare safety and immunogenicity of GP2015 and Enbrel® (EU-authorized) in patients with moderate to severe, active rheumatoid arthritis. http://www.clinicaltrialsregister.eu/ctr-search/search?query=2012-002009-23. Accessed 26 Mar 2016,

[86] Yi S, Kim SE, Park MK, Yoon SH, Cho JY, Lim KS (2012). Comparative pharmacokinetics of HD203, a biosimilar of etanercept, with marketed etanercept (Enbrel(R)): a double-blind, single-dose, crossover study in healthy volunteers. BioDrugs.

[87] ClinicalTrials.gov. Comparison of CHS-0214 to enbrel (Etanercept) in patients with rheumatoid arthritis (RA) (CHS-0214-02). http://clinicaltrials.gov/ct2/show/NCT02115750. Accessed 26 Mar 2016.

[88] Chung H, Ahn L, Choi Y, Shin S, Jang I, Yu K, et al. LBEC0101, an etanercept biosimilar, showed comparable tolerability and pharmacokinetic profiles to those of etanercept in healthy male volunteers. 115th Annual Meeting of the American Society for Clinical Pharmacology and Therapeutics; Atlanta, Georgia, USA; Clin Pharmacol Ther. 95:S5–S6.

[89] ClinicalTrials.gov. Study to compare the safety and pharmacokinetic characteristics of LBEC0101 25 mg with those of Enbrel®. http://clinicaltrials.gov/ct2/show/NCT01145950. Accessed 26 Mar 2016.

[90] ClinicalTrials.gov. A study to evaluate the long-term safety and efficacy of LBEC0101 in subjects with active rheumatoid arthritis despite methotrexate (MTX). http://clinicaltrials.gov/ct2/show/NCT02715908. Accessed 26 Mar 2016.

[91] Gu N, Kim BR, Kim BH, Kim JW, Song DH, Shin SG, et al. Comparative pharmacokinetics/tolerability of TuNEX and Enbrel® in healthy Korean volunteers. 111th Annual Meeting of the American Society for Clinical Pharmacology and Therapeutics; Atlanta, Georgia, USA; Clin Pharmacol Ther: 2010.

[92] Gu N, Yi S, Kim TE, Kim J, Shin SG, Jang IJ (2011). Comparative pharmacokinetics and tolerability of branded etanercept (25 mg) and its biosimilar (25 mg): a randomized, open-label, single-dose, two-sequence, crossover study in healthy Korean male volunteers. Clin Ther.

[93] • Cohen S, Pablos JL, Zhang N, Rizzo W, Muller G, Padmanaban D, et al. ABP 501 long-term safety/efficacy: interim results from an open-label extension study. American College of Rheumatology 2016; Washington DC, USA; 2016;68(suppl 10). http://acrabstracts.org/abstract/abp-501-long-term-safetyefficacy-interim-results-from-an-open-label-extension-study/. Accessed 17 May 2017. **Only published study to date of switching from adalimumab to ABP 501 in patients with RA. This study provides important efficacy, safety and immunogenicity data on switching**.

[94] Strober B, Foley P, Kaur P, Philipp S, Zhang N. Evaluation of efficacy and safety of ABP 501 in a phase 3 study in subjects with moderate to severe plaque psoriasis: 52-week results. American Academy of Dermatology 74th Annual Meeting; Washington DC, USA; J Am Acad Dermatol: 2016.

[95] Gooderham M, Constanzo A, Kaliaperumal A, Kaur P, Narbutt J, Spelman L, et al. Single transition from adalimumab to ABP 501: evaluation of immunogenicity in a phase 3 study in subjects with moderate to severe plaque psoriasis. American Academy of Dermatology 74th Annual Meeting; Washington DC, USA; J Am Acad Dermatol: 2016.

[96] • Weinblatt M, Baranauskaite A, Niebrzydowski J, Dokoupilova E, Zielinska A, Sitek-Ziolkowska K, et al. Sustained efficacy and comparable safety and immunogenicity after transition to SB5 (an adalimumab biosimilar) vs continuation of the adalimumab reference product in patients with rheumatoid arthritis: result of phase III study. European League Against Rheumatism 2016; London, UK; Ann Rheum Dis: 2016. **Only published study to date of switching from adalimumab to SB5 in patients with RA. This study provides important immunogenicity data on switching, although efficacy and safety data are lacking.**

[97] • Genovese MC, Weinblatt M, Keystone EC, Baranauskaite A, Cheong SY, Ghil J. Efficacy after transition to SB5 from reference adalimumab (Humira®) vs. continuation of SB5 or reference adalimumab by antibodies developed after transition from a SB5 phase III study. American College of Rheumatology 2016; Washington DC, USA: 2016. **Only published study to date of switching from adalimumab to SB5 in patients with RA. This study provides important immunogenicity data on switching, although efficacy and safety data are lacking.**

[98] EU Clinical Trials Register. A phase 3 randomized, double-blind, multicenter study to evaluate efficacy, safety, and immunogenicity of an adalimumab biosimilar (M923) and Humira® in subjects with moderate to severe chronic plaque-type psoriasis. http://www.clinicaltrialsregister.eu/ctr-search/trial/2015-001751-76/SK. Accessed 22 Apr 2016.

[99] • Yoo DH, Park W, Suh CH, Shim SC, Cons Molina F, Jeka S, et al. Efficacy and safety of switched CT-P10 from innovator rituximab compared to those of maintained CT-P10 in patients with rheumatoid arthritis up to 56 weeks. American College of Rheumatology 2015; San Francisco, California, USA; Arthritis Rheumatol: 2015. **Only published study to date of switching from rituximab to CT-P10 in patients with RA. This study provides important efficacy and safety data on switching, although immunogenicity data are lacking.**

[100] ClinicalTrials.gov. GP2013 treatment in patients with active rheumatoid arthritis, previously treated with Rituxan® or MabThera® (ASSIST-RT). http://clinicaltrials.gov/show/NCT02514772. Accessed 29 May 2016.

[101] Yoo DH, Racewicz A, Brzezicki J, Yatsyshyn R, Arteaga ET, Baranauskaite A (2016). A phase III randomized study to evaluate the efficacy and safety of CT-P13 compared with reference infliximab in patients with active rheumatoid arthritis: 54-week results from the PLANETRA study. Arthritis Res Ther.

[102] Park W, Yoo DH, Jaworski J, Brzezicki J, Gnylorybov A, Kadinov V (2016). Comparable long-term efficacy, as assessed by patient-reported outcomes, safety and pharmacokinetics, of CT-P13 and reference infliximab in patients with ankylosing spondylitis: 54-week results from the randomized, parallel-group PLANETAS study. Arthritis Res Ther.

[103] Takeuchi T, Yamanaka H, Tanaka Y, Sakurai T, Saito K, Ohtsubo H (2015). Evaluation of the pharmacokinetic equivalence and 54-week efficacy and safety of CT-P13 and innovator infliximab in Japanese patients with rheumatoid arthritis. Mod Rheumatol.

[104] Park W, Hrycaj P, Jeka S, Kovalenko V, Lysenko G, Miranda P (2013). A randomised, double-blind, multicentre, parallel-group, prospective study comparing the pharmacokinetics, safety, and efficacy of CT-P13 and innovator infliximab in patients with ankylosing spondylitis: the PLANETAS study. Ann Rheum Dis.

[105] Inman RD, Maksymowych WP (2010). A double-blind, placebo-controlled trial of low dose infliximab in ankylosing spondylitis. J Rheumatol.

[106] Braun J, Deodhar A, Dijkmans B, Geusens P, Sieper J, Williamson P (2008). Efficacy and safety of infliximab in patients with ankylosing spondylitis over a two-year period. Arthritis Rheum.

[107] Breban M, Ravaud P, Claudepierre P, Baron G, Henry YD, Hudry C (2008). Maintenance of infliximab treatment in ankylosing spondylitis: results of a one-year randomized controlled trial comparing systematic versus on-demand treatment. Arthritis Rheum.

[108] Gossec L, Le Henanff A, Breban M, Vignon E, Claudepierre P, Devauchelle V (2006). Continuation of treatment with infliximab in ankylosing spondylitis: 2-yr open follow-up. Rheumatology (Oxford).

[109] Braun J, Brandt J, Listing J, Zink A, Alten R, Burmester G (2005). Two year maintenance of efficacy and safety of infliximab in the treatment of ankylosing spondylitis. Ann Rheum Dis.

[110] Temekonidis TI, Alamanos Y, Nikas SN, Bougias DV, Georgiadis AN, Voulgari PV (2003). Infliximab therapy in patients with ankylosing spondylitis: an open label 12 month study. Ann Rheum Dis.

[111] Vencovský J, Sylwestrzak A, Leszczyński P, Porawska W, Baranauskaite A, Tseluyko V, et al. A phase III, randomized, double-blind clinical study comparing SB4, an etanercept biosimilar, with etanercept reference product (Enbrel®) in patients with moderate to severe rheumatoid arthritis despite methotrexate therapy (52-week results). American College of Rheumatology 2015; San Francisco, California, USA; Arthritis Rheum: 2015. doi:10.1136/annrheumdis-2015-eular.1220.

[112] Scheinberg M, Azevedo V (2016). Difference between enbrel and benepali treatment groups in ‘hepatobiliary disorders’. Ann Rheum Dis.

[113] Emery P, Vencovský J, Ghil J, Cheong SY, Rho YH. Difference between SB4 and reference etanercept in the hepatobiliary disorders not considered to be caused by SB4: response to letter by Scheinberg and Azevedo. Ann Rheum Dis. 2016;75:e65. doi:10.1136/annrheumdis-2016-210127. 10.1136/annrheumdis-2016-210127 27502892

[114] Yoo DH, Park W, Jeka S, Cons Molina F, Hrycaj P, Wiland P, et al. A randomized, controlled, multicenter, 2-arm, parallel-group, double-blind study to demonstrate the equivalence of CT-P10 to innovator rituximab with respect to pharmacokinetic profile in patients with rheumatoid arthritis. American College of Rheumatology 2013; San Diego, California, USA: 2013.

[115] Singh SC, Bagnato KM (2015). The economic implications of biosimilars. Am J Manag Care.

[116] Lee H (2014). Is extrapolation of the safety and efficacy data in one indication to another appropriate for biosimilars?. AAPS J.

[117] Flood J, Mihalik C, Fleming RR, Strober BE, Zucker DR, Burgoyne DS. The use of therapeutic interchange for biologic therapies. Manag Care. 2007;16:51–62. 17285813

[118] inVentiv Health Clinical. Elderly representation in clinical trials: not a gray area. http://www.inventivhealthclinical.com/resource-library-white-papers.htm. Accessed 10 Mar 2016.

[119] Chu R, Torstensson D, Pugatch M. Patient safety and comfort. The challenges of switching medicines. Stockholm Network. 2010. http://www.patients-rights.org/uploadimages/Patient_Safety_and_Comfort_The_Challenges_of_Switching.pdf. Accessed 31 Mar 2016.

