# Supplementary material for: Switching Between Reference Biologics and Biosimilars for the Treatment of Rheumatology, Gastroenterology, and Dermatology Inflammatory Conditions: Considerations for the Clinician
Source: Curr Rheumatol Rep. 2017 Jun 16;19(6):37. doi: 10.1007/s11926-017-0658-4 (PMC5486595; doi:10.1007/s11926-017-0658-4)
Supplement: Supplementary file 1 — (DOCX 13 kb) [file 11926_2017_658_MOESM1_ESM.docx]

Switching Between Reference Biologics and Biosimilars for the Treatment of Rheumatology, Gastroenterology, and Dermatology Inflammatory Conditions: Considerations for the Clinician

Current Rheumatology Reports

Robert Moots* · Valderilio Azevedo · Javier L. Coindreau · Thomas Dörner · Ehab Mahgoub · Eduardo Mysler · Morton Scheinberg · Lisa Marshall

* Corresponding Author: University of Liverpool, Liverpool, UK. rjmoots@liv.ac.uk

Online Resource 1:

Database searches

| **Search title** | **Search string(s)** |
| --- | --- |
| Inflammation | (biologic* OR biosimilar OR intended copy OR bio-equivalent) AND (switch* OR change OR convert OR conversion OR subsequent OR sequen* OR transition*) AND (anti-TNF OR TNF inhibitor OR anti-CD20 OR CD20 inhibitor OR anti-inflammatory) |
| Switching | (adalimumab OR etanercept OR infliximab OR rituximab) AND biosimilar AND (switch* OR transition* OR interchang*) |
| Etanercept | (Anbainuo OR AVG01 OR BX2922 OR BAX 2200 OR CHS-0214 OR CT-P05 OR davictrel OR DA-3853 OR DMB-3853 OR DWP422 OR GP2015 OR HD203 OR etanar OR etacept OR infinitam OR Intacept OR LBEC0101 OR tunex OR TNFcept OR PRX-106 OR Qiangke OR Reumatocept OR SB4 OR ENIA11 OR Yisaipu OR Benepali OR Erelzi OR Brenzys OR Etacept OR Intacept) AND (etanercept OR biosimilar) |
| Adalimumab | (ABP 501 OR BCD-057 OR BI695501 OR B0W050 OR CHS-1420 OR GP2017 OR LBAL OR M923 OR PF-06410293 OR ONS-3010 OR SB5 OR ZRC-3197) AND (adalimumab OR biosimilar) |
| Infliximab | (Inflectra OR Remsima OR ABP 710 OR BCD-055 OR CT-P13 OR NI-071 OR PF-06438179 OR SB2 OR BOW015 OR IFXBS) AND (infliximab OR biosimilar) |
| Rituximab | (ABP 798 OR BCD-020 OR CT-P10 OR GP2013 OR MK-8808 OR PF-05280586 OR SAIT101 OR TL011) AND (rituximab OR biosimilar) |
