# Supplementary material for: Switching Between Reference Biologics and Biosimilars for the Treatment of Rheumatology, Gastroenterology, and Dermatology Inflammatory Conditions: Considerations for the Clinician
Source: Curr Rheumatol Rep. 2017 Jun 16;19(6):37. doi: 10.1007/s11926-017-0658-4 (PMC5486595; doi:10.1007/s11926-017-0658-4)
Supplement: Supplementary file 2 — (DOCX 12 kb) [file 11926_2017_658_MOESM2_ESM.docx]

Switching Between Reference Biologics and Biosimilars for the Treatment of Rheumatology, Gastroenterology, and Dermatology Inflammatory Conditions: Considerations for the Clinician

Current Rheumatology Reports

Robert Moots* · Valderilio Azevedo · Javier L. Coindreau · Thomas Dörner · Ehab Mahgoub · Eduardo Mysler · Morton Scheinberg · Lisa Marshall

* Corresponding Author: University of Liverpool, Liverpool, UK. rjmoots@liv.ac.uk

Online Resource 2:

2016 Congresses searched for data on reference-biosimilar switching

| **Congress** |  |
| --- | --- |
| AAD | American Academy of Dermatology |
| ACR | American College of Rheumatology |
| ASCPT | American Society for Clinical Pharmacology and Therapeutics |
| BSR | British Society for Rheumatology |
| DDW | Digestive Disease Week |
| ECCO | European Crohn’s and Colitis Organization |
| EULAR | European League Against Rheumatism |
| SCR | Scandinavian Congress of Rheumatology |
| UEGW | United European Gastroenterology Week |
