# Supplementary material for: Switching Between Reference Biologics and Biosimilars for the Treatment of Rheumatology, Gastroenterology, and Dermatology Inflammatory Conditions: Considerations for the Clinician
Source: Curr Rheumatol Rep. 2017 Jun 16;19(6):37. doi: 10.1007/s11926-017-0658-4 (PMC5486595; doi:10.1007/s11926-017-0658-4)
Supplement: Supplementary file 3 — (DOCX 80 kb) [file 11926_2017_658_MOESM3_ESM.docx]

Switching Between Reference Biologics and Biosimilars for the Treatment of Rheumatology, Gastroenterology, and Dermatology Inflammatory Conditions: Considerations for the Clinician

Current Rheumatology Reports

Robert Moots* · Valderilio Azevedo · Javier L. Coindreau · Thomas Dörner · Ehab Mahgoub · Eduardo Mysler · Morton Scheinberg · Lisa Marshall

* Corresponding Author: University of Liverpool, Liverpool, UK. rjmoots@liv.ac.uk

Online Resource 3:

Key attributes and findings of switching studies of reference and biosimilar infliximab, etanercept, adalimumab, and rituximab

| **Study/Publication**  **(Biosimilar/ Proposed biosimilar)** | **Patient/subject population(s)** | **Efficacy** | **Safety** | **Immunogenicity** |
| --- | --- | --- | --- | --- |
| **Infliximab** | | | | |
| PLANETRA [[1](#_ENREF_1), [2](#_ENREF_2)]  (CT-P13) | 158 maintained on CT-P13  144 switched reference→CT-P13 | At study end (102 weeks), ACR20/50/70 response rates were comparable for the M and S groups:  ACR20: 71.7% (M) vs 71.8% (S)  ACR50: 48.0% (M) vs 51.4% (S)  ACR70: 24.3% (M) vs 26.1% (S)  Response rates at week 102 for patients who switched treatments were similar to those observed at the pre-switch time point (wk54):  ACR20: 77.5% (INF, wk54) vs 71.8% (CT-P13, wk102)  ACR50: 50.0% (INF, wk54) vs 51.4% (CT-P13, wk102)  ACR70: 23.9% (INF, wk54) vs 26.1% (CT-P13, wk102)  No notable differences in other efficacy endpoints were observed | The proportion of patients who experienced ≥1 TEAE was comparable for the M and S groups:  Pre-switch (DB): 63.5% (M) vs 62.2% (S)  Post-switch (OLE): 53.5% (M) vs 53.8% (S)  The proportion of patients experiencing a study-related TEAE was comparable for the M and S groups:  Pre-switch (DB): 35.2% (M) vs 35.7% (S)  Post-switch (OLE): 22.0% (M) vs 18.9% (S) | The proportion of patients with ADAbs was similar between the M and S groups at each time point in the DB and OLE study phases  Wk54: 49.1% (M) vs 48.3% (S); p=0.91  Wk102:40.3% (M) vs 44.8% (S); p=0.48 |
| PLANETAS [[3](#_ENREF_3), [4](#_ENREF_4)]  (CT-P13) | 88 maintained on CT-P13  86 switched reference→CT-P13 | At study end (102 weeks), ASAS20/40 and ASAS PR were comparable for the maintenance (M) and switch (S) groups:  ASAS20: 80.7% (M) vs 76.9% (S); p=0.51  ASAS40: 63.9% (M) vs 61.5% (S); p=0.67  ASAS PR: 19.3% (M) vs 23.1% (S); p=0.28  Response rates at Week 102 for patients who switched treatments were similar to those observed at the pre-switch time point (wk54):  ASAS20: 75.6% (wk54) vs 76.9% (wk102)  ASAS40: 53.5% (wk54) vs 61.5% (wk102)  ASAS PR: 17.4% (wk54) vs 23.1% (wk102)  No notable differences in other efficacy endpoints were observed | The proportion of patients who experienced ≥1 TEAE was comparable for the M and S groups:  Pre-switch (DB): 70.0% (M) vs 61.9% (S)  Post-switch (OLE): 48.9% (M) vs 71.4% (S)  The proportion of patients experiencing a study-related TEAE was comparable for the M and S groups:  Pre-switch (DB): 44.4% (M) vs 41.7% (S)  Post-switch (OLE): 22.2% (M) vs 39.3% (S) | Immediately prior to switch and at study end, the proportion of patients with ADAbs was comparable for the M and S groups, with no increase in incidence from 54-102 weeks  Wk54: 22.2% (M) vs 26.2% (S); p=0.60  Wk102: 23.3% (M) vs 27.4% (S); p=0.60 |
| Tanaka et al, Mod Rheumatol 2016 Sept 1 (epub) [[5](#_ENREF_5)]  (CT-P13) | 38 maintained on CT-P13  33 switched reference→CT-P13 | 55 patients (32 in the maintenance group and 23 in the switch group) continued the study treatment through Week 134  In the maintenance group:  ACR response rates tended to show a slight improvement from the initiation of extension study through Week 134  The proportions of patients achieving ACR20, ACR50 and ACR70, respectively, were 78.4% (29/37 patients), 70.3% (26/37 patients) and 54.1% (20/37 patients) at Week 134  The mean scores (mean ±SD) of DAS28 (ESR) at Weeks 62, 110 and 134 were 3.220±1.328, 3.180±1.693, and 3.166 ±1.533, and the mean changes (mean ±SD) from baseline were –2.707±1.589, –2.747±1.727, and –2.761±1.613, respectively  In the switch group:  ACR response rates tended to show a slight improvement from the initiation of extension study through Week 134  The proportions of patients achieving ACR20, ACR50 and ACR70, respectively, were 62.5% (20/32 patients), 53.1% (17/32 patients) and 40.6% (13/32 patients) at Week 134  The mean scores (mean ±SD) of DAS28 (ESR) at Weeks 62, 110 and 134 were 3.882±1.496, 3.979±1.721 and 3.955±1.751, and the mean changes (mean ±SD) from baseline were –2.036 ±1.305, –1.940±1.664 and –1.964±1.711, respectively | In both the maintenance and switch group, there were no notable differences between the phase I/II study and the extension study in AEs, adverse drug reactions (ADRs), SAEs, infection, and infusion-related reactions. | In the maintenance group, positive rates of ADAb at Week 110 and Week 134 were 11.8% (4/34 patients) and 15.6% (5/32 patients). Twenty-five of 26 patients who were negative for ADAb at the initiation of the extension study remained negative throughout the study, and 5 of 12 patients who were positive for ADAb became negative during the study  In the switch group, positive rates for ADAb at Week 110 and 134 were 21.7% (5/23 patients) and 17.4% (4/23 patients). Sixteen of 17 patients who were negative for ADAb at the initiation of the extension study remained negative throughout the study, and 5 of 16 patients who were positive for ADAb became negative during the study |
| Yazici et al, 2016, ACR abstract 2240 [[6](#_ENREF_6)]  (CT-P13) | 2870 maintained on reference  148 switched reference→CT-P13 | A high percentage of the patients on CTP-13 discontinued therapy (82% vs 38% on INF)  Of the CT-P13 patients who discontinued, 74% switched to another biologic and 94% of these returned to INF | No outcomes reported | No outcomes reported |
| DANBIO [[7-11](#_ENREF_7)]  (CT-P13) | 792 switched reference→CT-P13 | Disease activity and disease flare rates remained largely unchanged 3 months prior to versus after the switch  Change from 3 months’ pre-switch→switch versus switch→3 months’ post-switch (median and IQR):  RA, PsA:  DAS28: 0.0 (-0.3-0.5) vs 0.0 (–0.3-0.3) [p=0.4]  HAQ: 0.0 (0.0-0.1) vs 0.0 (0.0-0.1) [p=0.9]  CRP: 0 (–1-2) vs 0 (–2-3) [p=0.09]  Patient global VAS: 0 (–7-8) vs 0 (–7-9) [p=0.2]  AxSpA:  BASDAI: 0 (–4-5) vs 0 (–3-6) [p=0.5]  CRP: 0 (–1-1) vs 0 (–2-2) [p=0.4]  Patient global VAS: 1 (–6-9) vs –1 (–8-4) [p=0.4]  ASDAS: 0 (–3-4) vs 0 (–4-2) [p=0.6]  The proportion of patients with disease flare was similar pre- versus post-switch:  RA, PsA (ΔDAS28≥0.6): 25% vs 21% [p=0.4]  RA, PsA (ΔDAS28≥1.2): 10% vs 10% [p=0.8]  AxSpA: 2% vs 2% [p=1.0]  51 patients discontinued because of LoE | 34 patients discontinued because of AEs | 2/81 patients with low baseline ADAb had medium-high ADAb at follow-up  3/15 pts with medium-high baseline ADAb had low ADAb at follow-up  ADAb remained stable between baseline and follow-up in 91/96 (95%) of patients |
| Holroyd et al, 2016, BSR abstract O52 [[12](#_ENREF_12)]  (CT-P13) | 56 switched reference→CT-P13 | For RA, the mean DAS28 was 3.3 prior to and 4.1 after switching.  For AS, the mean BASDAI was 3.5 prior to and after the switch.  3 patients discontinued infliximab treatment in the year before the switch because of inefficacy; 3 discontinued in the 6 months after the switch because of inefficacy  Four patients switched back to reference because of inefficacy | 7 patients discontinued infliximab treatment in the year before the switch because of AEs; 1 discontinued in the 6 months after the switch because of AEs  One patient switched back to reference because of AEs | No outcomes reported |
| Benucci et al, Immunol Res 2016 Jul 23 (epub) [[13](#_ENREF_13)]  Batticciotto et al 2016, EULAR abstract SAT0381 [[14](#_ENREF_14)]  Batticciotto et al 2016, ACR abstract 271 [[15](#_ENREF_15)]  (CT-P13) | 41 switched reference→CT-P13 | After 6 months of bINX therapy, there were no statistical differences in the median  BASDAI (2.73±1.5 vs 2.6±1.3, p=0.27),  BASFI (2.34±1.3 vs 2.17±1.2, p=0.051),  ASDAS-CRP (1.35±0.3 vs 1.28±0.2, p=0.24),  DAS28-CRP (2.66±0.67 vs 2.67±0.35, p=0.92),  MASES (0.35±0.7 vs 0.17±0.4, p=0.08),  VAS pain scores (18±14.7 vs 16,7±11.3, p=0.55),  After 6 months of bINF therapy, median duration of morning stiffness was significantly shorter (7.2±6.9 vs 5.8±6 p=0.02) | During the first 6 months of bINX treatment, very few patients experienced an AE, and there was no significance difference from the AEs recorded during the 6 months before the switch (p=1.0). AEs led to treatment discontinuation only in 1 patient (3%) | There was no change in anti-INX ADAb levels before and after switch (27.76±17.13 vs 27.27±17.28 ng/mL, p=0.98) |
| Sokka et al, 2015, EULAR abstract SAT0174 [[16](#_ENREF_16)]  Nikiphorou et al, 2015 [[17](#_ENREF_17)]  (CT-P13) | 39 switched reference→CT-P13 | There was no significant difference in patient-reported outcomes for reference INF and CT-P13 | Latent tuberculosis re-activation was seen in 1 patient following the second CT-P13 infusion. In another patient, new onset neurofibromatosis was diagnosed after the second CT-P13 infusion  Six patients discontinued CT-P13 for subjective reasons without objective deterioration of disease | Three patients tested positive for ADAbs before CT-P13 was given. Immunogenicity on CT-P13 was not assessed |
| Abdalla et al 2016, EULAR abstract THU0120 [[18](#_ENREF_18)]  (CT-P13) | 34 switched reference→CT-P13 | No difference in efficacy in the first 6 months of therapy after the switch vs before  However, reported pain and CRP values were significantly higher during the longer follow-up period (p value 0.028, 0.001, respectively). | No difference in safety in the first 6 months of therapy after the switch vs before | No outcomes reported |
| Malaiya et al 2016 BSR abstract 158 [[19](#_ENREF_19)]  (CT-P13) | 30 switched reference→CT-P13 | 1/30 patients was subjectively worse after the first 3 infusions and switched back to reference | No outcomes reported | No outcomes reported |
| BIO-SWITCH [[20](#_ENREF_20)]  (CT-P13) | 192 switched reference→CT-P13 | 44/192 (23%) patients discontinued CT-P13 during 6  months follow-up; 34 patients restarted innovator infliximab, 7 patients switched to another biological (2 adalimumab, 3 etanercept, 1 golimumab, 1 rituximab) and 3 patients maintained biological-free  35/192 (18%) discontinued CT-P13 treatment in the first 6 months follow-up owing to inefficacy  In RA and PsA patients mean DAS28-CRP remained stable from month 0 to 6: 2.19 [SD 0.89] to 2.22 [SD 0.84] (p=0.51).  In SpA patients, mean BASDAI increased from 3.8 to 4.3 (change +0.5, 95% CI 0.12-0.89, p=0.01).  CRP levels at baseline (median 1.5 mg/L [p25-p75:0-5]) and 6 months (median 1.0 mg/L [p25-p75: 0-5]) were not statistically different (p=0.60). | 23/192 (12%) discontinued CT-P13 treatment in the first 6 months’ follow-up owing to AEs  2/192 (1%) discontinued CT-P13 treatment in the first 6 months’ follow-up owing to infusion reactions  Most frequently reported AEs resulting in biosimilar discontinuation were fatigue (n=10), malaise (n=5) and headache (n=3). No serious AEs occurred | ADAbs were detected in 15/32 (47%) patients at baseline and in 10/26 (38%) patients after 6 months |
| Buer et al, 2016 [[21](#_ENREF_21)] | 143 switched reference→CT-P13 | No significant changes in disease activity (CD: HBI; UC: PMS) were observed post-switch  The proportion of patients in clinical remission was  CD: 87% (wk0) → 81% wk 26 (HBI ≤4)  UC: 88% (wk0) → 95% wk 26 (PMS <2)  N=69 (70%) of CD patients and 32 (73%) of UC patients stayed in remission throughout the follow-up  Median change in HGBI scores was 0 (–24 to 9) (switch→3 months) and 0 (–8 to 10) (3→6 months)  Median change in PMS was 0 (–1 to 7) (switch →3 months) and 0 (–2 to 5) (3 →6 months)  No statistically significant changes in biochemical markers of inflammation (CRP, Hgb, FC) were detected  No statistically significant changes in CRP, Hgb or FC were observed over time or at any selected time point, neither before nor after switch | AEs were observed in 3 patients with UC and 14 patients with CD  2 patients discontinued due to AEs  No patient experienced more than one AE  Among 750 infusions, only 5 (0.7%) infusion reactions (all in 2 patients) were observed | ADAbs were detected in 5 patients  2 patients (males, 1 UC and 1 CD) had detectable ADAbs at baseline that were not detectable later during follow-up  All patients with detectable ADAbs after switch had CD  4 patients had low ADAb levels of (10-30 AU/L)  1 patient had moderate ADAb levels (30-80 AU/L)  In all these patients, ADAbs were transient and patients continued treatment during the 6-month observation period |
| Bettey et al, 2016, ECCO abstract DOP019 [[22](#_ENREF_22)]  (CT-P13) | 134 switched reference→CT-P13 | No outcomes reported | Patients reported a similar incidence of expected side effects before and after the switch | No outcomes reported |
| Jung et al, 2015 [[23](#_ENREF_23)]  Jung et al, 2015, ECCO abstract P540 [[24](#_ENREF_24)]  (CT-P13) | 36 switched reference→CT-P13  74 anti-TNFα−naïve started CT-P13 | In patients with CD who switched to CT-P13, 25/27 (92.6%) maintained a similar efficacy to reference infliximab over 54 weeks, and 2 (7.4%) discontinued due to lack of efficacy  In patients with UC who switched to CT-P13, 6/9 (66.7%) maintained a similar efficacy to reference infliximab over 54 weeks, and 3 (33.3%) discontinued due to either lack of efficacy (n=1), patient preference (n=1) or AE (n=1) | 1 patient who switched to CT-P13 discontinued treatment because of skin rash and arthralgia | No outcomes reported |
| Smits et al, 2016, ECCO abstract DOP030 [[25](#_ENREF_25)]  Smits et al, 2016 [[26](#_ENREF_26)]  (CT-P13) | 83 switched reference→CT-P13 | At study end (16 weeks), disease activity and proportion of patients in clinical remission was similar to that observed at time of switch (wk0)  HBI (CD patients): 3.0 (wk0) vs 3.0 (wk16); p=0.41  SCCAI (UC patients): 1.5 (wk0) vs 2.0 (wk16); p=0.17  60% (wk0) vs 67% (wk16) of CD patients were in clinical remission; p=0.48  73% (wk0) vs 62% (wk16) of UC patients were in clinical remission; p=0.25  80% of CD patients maintained remission from wk0→wk16  84% of UC patients maintained remission from wk0→wk16  Inflammatory biomarkers did not significantly change from wk0→wk16:  Median FCP was 52.0 mg/kg (wk0) vs 43.0 mg/kg (wk16); p=0.70  Median CRP was 1.0 mg/L (wk0) vs 1.0 mg/L (wk16); p=0.42  No notable differences in other efficacy endpoints were observed | 24/82 (29.3%) patients reported AEs  Suspected adverse reactions occurred in 6 (7%) patients with 5 able to continue therapy  5 patients discontinued CT-P13 treatment: 3 because of high ADAb and undetectable TL measured at week 0; 1 patient because of arthralgia; and 1 patient who moved abroad | The proportion of patients with ADAbs was 7/83 (8.4%); this included 5 patients who had pre-existing detectable ADAb levels at study baseline. An additional 2 patients developed detectable levels of ADAbs during follow-up |
| Guerra Veloz et al, 2016, ECCO abstract P452 [[27](#_ENREF_27)]  (CT-P13) | 71 switched reference→CT-P13  4 INF-naïve started CT-P13 | At the time of the switch, 81.6% of 71 patients were in remission and 28% were not in remission  At 3 months 62 patients were analyzed (60 switch and  2 naïve):  72.3% of patients were in remission  23.1% of patients not in remission when treatment was switched were in remission  1 naïve patient reached remission  At 6 months 31 patients were analyzed (29 switch and 2 naïve):  70% of patients continued in remission  33.3% of patients not in remission when treatment was switched reached remission.  The 2 naïve patients were in remission | In 5 patients (6.6%) mild AEs were  noted (2 abdominal pains, 2 headaches, and 1 tongue paresthesia) | No outcomes reported |
| Kolar et al, 2016, ECCO abstract DPO032 [[28](#_ENREF_28)]  Kolar et al, 2016, UEGW abstract P1410 [[29](#_ENREF_29)]  (CT-P13) | 74 switched reference→CT-P13 | The proportion of patients in clinical remission was stable from time of switch (wk0; 72%) to study end (wk24; 78%)  Inflammatory biomarkers did not significantly change from wk0→wk24:  CRP was 4.3±8.0 mg/L (wk0) vs 3.6±4.5 mg/L (wk24); p=0.78  FCP was 135±153 μg/g (wk0) vs 226±297 μg/g (wk24); p=0.44 | The frequency and type of AEs were similar to those observed during treatment with reference INF  No patients experienced infusion reactions | No increase in immunogenicity was observed from time of switch to study end:  IFX TL: 3.4±3.8 μg/mL (wk0) vs 3.8±3.3 μg/mL (wk24); p=0.23  ADAb positivity: 9.5% (wk0) vs 10.0% (wk24); p=0.79 |
| Guerra Veloz et al, 2016, ECCO abstract P600 [[30](#_ENREF_30)]  (CT-P13) | 31 switched reference→CT-P13  9 INF-naïve started CT-P13 | 23 patients were in remission when switch was carried out, 8 were not in remission, and 9 were naïve to anti-TNF  At 3 months 34 patients were analyzed (27 switch and 7 naïve):  73.7% of patients in remission when treatment was switched, were in remission at 3 months  All naïve patients reached remission at that time  At 6 months, 16 patients were analyzed (15 switch and 1 naïve):  70% of patients continued in remission  23% of patients not in remission when treatment was switched reached remission  No differences were found between the group of switched patients that continued in remission and the group that was not in remission | In 2 patients (5%) mild AEs were noted (headaches) | No outcomes reported |
| Jarzebicka et al, 2015; ECCO abstract P295 [[31](#_ENREF_31)]  Kierkus, 2015; AGA abstract Tu1081 [[32](#_ENREF_32)]  Sieczkowska et al, 2016 [[33](#_ENREF_33)]  Sieczkowska et al 2016, ECCO abstract P617 [[34](#_ENREF_34)]  (CT-P13) | 32 switched reference→CT-P13 | Mean disease activity (PCDAI) and laboratory values were comparable at the penultimate INF, last INF, first CT-P13 (switch) and second CT-P13 infusions (respectively):  PCDAI: 7 (wk-16) vs 5.9 (wk-8) vs 8.5 (wk0) vs 7.5 (wk8)  CRP: 1.5 (wk-16) vs 1.2 (wk-8) vs 1.2 (wk0) vs 0.6 (wk8)  ESR: 13 (wk-16) vs 15 (wk-8) vs 14 (wk0) vs 13 (wk8) | The occurrence of sporadic mild AEs did not differ significantly when measured before and after switching, and was consistent with the INF molecule safety profile  At weeks 16, 24 and 32 after switch, 16, 5 and 4 patients were evaluated with no disease flare or unexpected AEs | 16 patients were assessed for ADAbs  14/16 patients had therapeutic levels (>1.5 μg/mL) of reference biologic and 1/16 had sub-therapeutic levels at the time of switch  7/16 patients tested positive for anti-TNFα ADAbs at the time of switching (>2 ng/mL)  15 patients were assessed for ADAbs after switching  15/15 patients had therapeutic levels of CT-P13  4/15 had ADAbs (>2 ng/mL) |
| Hlavaty et al 2016, ECCO abstract P655 [[35](#_ENREF_35)]  (CT-P13) | 12 switched reference→CT-P13  13 infliximab-naïve started CT-P13 | Of 12 patients (10 CD; 2 UC) who switched from IFX:  100% had sustained clinical response at week 24  87.5% (6/7 CD; 1/1 UC) had sustained clinical response at week 32  75% (5/7 CD; 1/1 UC) had sustained clinical response at week 48  Of 13 patients (9 CD; 4 UC) who initiated CT-P13:  84% (7/9 CD; 4/4 UC) achieved clinical remission by week 14  Sustained clinical response was maintained in 85% (3/3 CD; 3/4 UC) at week 30 | 4 CD patients discontinued CT-P13 therapy because of injection reactions, psoriasiform skin rash, or loss of response | No outcomes reported |
| Kang et al, 2014; ECCO abstract P565 [[36](#_ENREF_36)]  Kang et al, 2015 [[37](#_ENREF_37)]  (CT-P13) | 9 switched reference→CT-P13  8 anti-TNFα−naïve started CT-P13 | 8/9 patients who switched showed a similar clinical outcome compared with reference INF | 8/9 patients experienced no adverse drug reactions | No outcomes reported |
| PROSIT-BIO [[38](#_ENREF_38)]  Fiorino et al 2016, ECCO abstract P544 [[39](#_ENREF_39)]  (CT-P13) | 93 switched reference→CT-P13  217 anti-TNFα naïve started CT-P13  87 previously exposed to a biologic | After a median follow-up of 6 months, the proportion of patients (CD/UC) with treatment response, primary failure and loss of response was similar for patients who switched, were anti-TNFα -naïve and previously exposed:  Response: 94% switched, 92% naïve, 91% previously exposed  Primary failure: 5% switched, 8% naïve, 5% previously exposed  Loss of response: 12% switched, 1% naïve, 5% previously exposed; p<0.001 for switched vs naïve and previously exposed patients  Efficacy of CT-P13 was evaluated over 54 weeks in terms of clinical response/clinical remission in CD (CDAI<100/CDAI<150) and UC (Mayo <30%/Mayo ≤2) patients:  CD, clinical response: 90.6% (wk8), 95.5% (wk30), 87.5% (wk54)  CD, clinical remission: 84.4% (wk8), 77.3% (wk30), 75.0% (wk54)  UC, clinical response: 89.5% (wk8), 91.3% (wk30), 100% (wk54)  UC, clinical remission: 52.6% (wk8), 60.9% (wk30), 58.3% (wk54) | Safety was comparable across the patient groups | No outcomes reported |
| Park SH et al, 2015 [[40](#_ENREF_40)]  (CT-P13) | No CT-P13 maintenance group  60 switched reference→CT-P13  113 anti-TNFα-naïve started CT-P13 | For patients who switched:  25/31 (80.6%) CD patients achieved or maintained remission through wk2-wk30; 18 were in remission at switching  27/31 (87.1%) CD patients did not experience disease worsening and were considered to have disease control  2/4 (50.0%) FCD patients maintained remission; 3/4 (75.0%) maintained disease control (wk2-wk30)  5/11 (45.5%) UC patients achieved or maintained remission through wk2-wk30; 3 were in remission at switching  No patients experienced disease worsening, and all 11 patients were considered to have disease control | There were no meaningful differences observed in the proportion of patients experiencing TEAEs in the anti-TNFα-naïve group compared with the switch group:  In patients receiving 5 mg/kg CT-P13, 17 (15.0%) of naïve and 7 (11.7%) of switch patients had a TEAE  In patients receiving >5 mg/kg CT-P13, 10 (8.8%) of naïve and 4 (6.7%) of switch patients had a TEAE | No outcomes reported |
| Dapavo et al J Am Acad Dermatol 2016 Jul 26 (epub) [[41](#_ENREF_41)]  (CT-P13) | 30 switched reference→CT-P13  5 anti-TNF-naïve started CT-P13 | In the switch group, PASI and VAS scores were not significantly different (p>0.05) before the switch to the biosimilar and at the end of the observation period  In the INF-naïve group, 4/5 patients reached 75% improvement from baseline PASI score at Week 10 | During follow-up, 1 patient developed herpes zoster and his symptoms resolved completely after a 7-day course of systemic valacyclovir hydrochloride and paracetamol. No other AEs were observed | No outcomes reported |
| NOR-SWITCH [[42](#_ENREF_42), [43](#_ENREF_43)] | 241 maintained treatment with reference  240 switched reference→CT-P13 | Disease worsening occurred in 26.2% and 29.6% of patients in the INF and CT-P13 arms, respectively. The 95% confidence interval of the adjusted treatment difference (–4.4%) for the PPS population was –12.7 to 3.9 which was within the pre-specified non-inferiority margin of 15%  Disease worsening (n, %) for maintenance and switch was also reported by diagnosis:  RA: 11 (36.7%) vs 9 (30.0%)  SpA: 17 (39.5%) vs 14 (33.3%)  PsA: 7 (53.8%) vs 8 (61.5%)  CD: 14 (21.2%) vs 23 (36.5%)  UC: 3 (9.1%) vs 5 (11.9%)  PsO: 1 (5.9%) vs 2 (12.5%)  Changes in the generic disease variables and disease-specific measures (mean, SD) from baseline to study end were similar for both maintained and switched patients  PGA: 0.09 (1.62) vs 0.11 (1.56)  PtGA: 0.43 (1.87) vs 0.30 (2.20)  ESR (log): 0.019 (0.254) vs 0.006 (0.308)  CRP (log): 0.020 (0.345) vs 0.023 (0.419)  BASDAI (SpA): 0.25 (1.01) vs –0.15 (1.38)  ASDAS (SpA): 0.07 (0.59) vs –0.19 (0.67)  DAS28 (RA, PsA): 0.30 (0.98) vs 0.08 (0.93)  CDAI (RA, PsA): 1.51 (5.54) vs 0.67 (3.94)  SDAI (RA, PsA): 1.56 (5.67) vs 0.69 (4.41)  HBI (CD): 0.26 (2.35) vs 0.49 (3.15)  PMS (UC): 0.09 (1.28) vs –0.17 (1.68)  Log FC (UC, CD): 0.035 (0.506) vs 0.096 (0.477)  PASI (PsO): –0.50 (1.88) vs –0.44 (1.87) | Frequencies of reported AEs including infusion reactions were similar in the INF (maintenance) and CT-P13 (switch) patients, respectively (FAS) (data not presented) | Incidence of ADAbs was 17 (7.1%) and 19 (7.9%) in the INF (maintenance) and CT-P13 (switch) patients, respectively (FAS) |
| Gentileschi et al Expert Opin Biol Ther 2016;16:1311-2 [[44](#_ENREF_44)]  (CT-P13) | 23 switched reference→CT-P13 | 7/23 (30.4%) patients suffered relapse after a mean of 1.71 months on CT-P13; this lead to improvements in 5/7 of these patients  9/23 had no changes in response to treatment  7/23 were monitored for AEs | No AEs noted | No outcomes reported |
| Smolen et al 2016, EULAR abstract FRI0162 [[45](#_ENREF_45)]  Smolen et al 2016, ACR abstract 2596 [[46](#_ENREF_46)]  (SB2) | 201 received SB2 throughout  101 received reference throughout  94 switched reference→SB2 | Efficacy was sustained and comparable between the treatment groups | The safety profile during the transition period was comparable between INF/SB2, INF/INF and SB2/SB2  The incidence of AEs during the transition period was 36.2% in INF/SB2, 35.6% in INF/INF, and 40.3% in SB2/SB2. The incidence of infusion-related reaction during the transition period was 3.2%, 2.0% and 3.5%, respectively | Among the patients with overall negative ADAb results pre-switch, ADAbs newly developed in 14.6% (6/41) in INF/SB2, 14.9% (7/47) in INF/INF and 14.1% (11/78) in SB2/SB2 |
| Hamanaka et al 2016, ECCO abstract P329 [[47](#_ENREF_47)]  (Infliximab NK®/ IFXBS) | 3 switched reference→ IFXBS  17 infliximab-naïve started IFXBS | Amongst the 3 switched patients, CRP was slightly increased in 1 patient with CD, but all maintained remission at Week 24  Amongst the patients who received induction therapy:  5 (46%) UC and 5 (83%) of CD patients achieved remission at Week 6  4 (80%) UC and 3 (100%) CD patients achieved remission at Week 22 | Infusion reaction occurred in 1 patient who received induction therapy | No outcomes reported |
| Diaz Hernandez et al 2016, ECCO abstract P449 [[48](#_ENREF_48)]  (Unspecified) | 72 switched reference→ biosimilar | 62/72 patients were in clinical remission at the time of the switch  62/72 (86%) patients were in clinical remission at 6 months  3 patients had treatment withdrawn for primary (1) or secondary (2) non-response | After switch:  5 reports of AEs  5 patients reported disease flare  2 patients required surgery | No outcomes reported |
| **Etanercept** | | | | |
| Emery et al 2016, EULAR abstract THU0150 [[49](#_ENREF_49)]  Emery et al 2016, ACR abstract 603 [[50](#_ENREF_50)]  (SB4) | 126 maintained treatment with SB4  119 switched reference→ SB4 | Efficacy was sustained and comparable between maintained (M) and switched (S) patients up to Week 100:  LDA based on:  DAS28: 49.2% (M) vs 54.8% (S)  SDAI: 33.3% (M) vs 38.3% (S)  CDAI: 30.9% (M) vs 40.0%  Remission based on:  DAS28: 30.3% (M) vs 34.8% (S)  SDAI: 30.9% (M) vs 33.9% (S)  CDAI: 32.5% (M) vs 28.7% (S)  ACR20/50/70 responses:  ACR20: 77.9% (M) vs 79.1% (S)  ACR50: 59.8% (M) vs 60.9% (S)  ACR70: 42.6% (M) vs 41.7% (S) | Safety was comparable between maintained and switched patients:  60 (47.6%) maintained and 58 (48.7%) switched patients reported ≥1 TEAE  6 (4.8%) maintained and 2 (1.7%) switched patients reported ≥1 SAE | Incidence of ADAbs post-switch point was 0.8% for maintained patients and 0.9% for switched patients |
| Lee Y et al, 2016 [[51](#_ENREF_51)]  Lee YJ et al, 2016, EULAR abstract SAT0176 [[52](#_ENREF_52)]  (SB4) | Three study populations:  Population A:  23, single dose SB4→ single dose EU-sourced ETN  23, single dose EU-sourced ETN→ single dose SB4  Population B:  23, single dose SB4→ single dose US-sourced ETN  23, single dose US-sourced ETN→ single dose SB4  Population C:  23, US-sourced ETN → single dose EU-sourced ETN  23, single dose EU-sourced ETN→ single dose US-sourced ETN | Pharmacokinetic analysis showed SB4 to be equivalent to EU- or US-sourced etanercept (AUC_∞_ and C_max_) | The safety profile of SB4 was similar to EU- or US-sourced etanercept | No outcomes reported |
| EGALITY [[53](#_ENREF_53)]  (GP2015) | 150 maintained treatment with GP2015  151 maintained treatment with reference  100 switched GP2015→reference (3 switches)  96 switched reference→GP2015 | PASI 50/75/90 response rates were similar for patients who maintained treatment (combined reference and GP2015) and those who switched treatments | Safety profiles of GP2015 and reference were similar and not affected by switching  Incidence of AE of special interest was higher for continued GP2015 vs continued ETN (11.0% vs 4.7%); and for switched GP2015 (11 [11.0%]) vs switched ETN (5 [5.2%]) groups | Pre-switch, 5 (1.9%) patients receiving reference tested positive for ADAbs  During the period where switching was occurring, there was no additional seroconversion  In the period following the switching, ADAbs were detected in 1 (1.1%) patient switching from reference to GP2015 |
| Afonso et al 2016, EULAR abstract THU0145 [[54](#_ENREF_54)]  (GP2015) | 54 received a single dose of  1) reference→ GP2015, or  2) GP2015→ reference | Pharmacokinetic analysis showed GP2015 to be equivalent to reference (C_max_, AUC_0-tlast_ and AUC_0-inf_) | The most common AEs were:  Neutropenia [GP2015, n=7 (13%); reference, n=8 (14.8%)],  Headache [GP2015, n=5 (9.3%); reference, n=5 (9.3%)]  Nasopharyngitis [GP2015, n=4 (7.4%); reference, n=4 (7.4%)]. | 3 subjects in the treatment sequence GP2015→reference had low titer non-neutralizing ADAbs at the 28-day follow-up |
| **Adalimumab** | | | | |
| Cohen et al 2016, ACR abstract 616 [[55](#_ENREF_55)] | 229 maintained on ABP 501  237 switched reference→ABP 501 | Efficacy was similar in patients who transitioned from adalimumab compared with patients who continued on ABP 501 | The rates of TEAEs were similar between patients with single transition from adalimumab and those who  continued on ABP 501 | The rates of ADAbs were similar between patients with single transition from adalimumab and those who continued on ABP 501 |
| Strober et al 2016, AAD abstract 2957 [[56](#_ENREF_56)]  Gooderman et al 2016 [[57](#_ENREF_57)]  (ABP 501) | 152 maintained on ABP 501  77 switched reference→ ABP 501  79 maintained on reference | No outcomes reported | No outcomes reported | Incidence of ADAbs was similar and comparable between ABP 501 and adalimumab before and after re-randomization. Single transition to ABP 501 did not result in altered or increased immunogenicity over 52 weeks  At week 16 (re-randomization, pre-switch), ADAb incidence was:  63.6% for reference  55.2% for ABP 501  At week 20 (post-switch), ADAb incidence was:  69.6% for maintained reference  60.5% for maintained ABP 501  66.2% for switched patients  At week 52 (study end), ADAb incidence was:  74.7% for maintained reference  68.4% for maintained ABP 501  72.7% for switched patients |
| Weinblatt et al 2016, EULAR abstract FRI0161 [[58](#_ENREF_58)]  Weinblatt et al 2016, ACR abstract 604 [[59](#_ENREF_59)]  Genovese et al 2016, ACR abstract 622 [[59](#_ENREF_59)]  (SB5) | 254 maintained on SB5 (S/S)  125 switched reference→ SB5 (A/S)  129 maintained on reference (A/A) | ACR20 responses were comparable between the 3 groups at Week 52 (S/S: 76.9%; A/S: 81.1%; A/A: 71.2%)  Change in modified total Sharp score was comparable between the 3 groups at Week 52 (mean change for S/S: 0.17; A/S: 0.25; A/A: 0.50)  Other efficacy endpoints were also comparable between the 3 treatment groups | The safety profile during the transition period was comparable across treatment groups. The proportion of patients with ≥1 TEAE after switching was  S/S: 32.2%  A/S: 37.6%  A/A: 33.1%  2 A/A patients reported injection site reactions | Incidence of ADAbs after switching was  S/S: 15.7%  A/S: 16.8%  A/A: 18.3% |
| **Rituximab** | | | | |
| Yoo et al 2015, ACR abstract 1675 [[60](#_ENREF_60)]  (CT-P10) | 58 maintained on CT-P10  29 switched reference rituximab→CT-P10 | The DAS28-CRP and ESR improvement at Week 24 after the last CT-P10 infusion were similar in the 2 treatment groups;  DAS28-CRP: –2.2 for both groups; p=0.95  DAS28-ESR: –2.7 for maintained CT-P10 group and –-2.4 for switched CT-P10 group; p=0.57 | The proportion of patients experienced ≥1 AE or SAE was comparable between maintained and switched CT-P10 groups:  AEs: 23.7% (M) vs 20.0% (S)  SAEs: 2.6% (M) vs 5.0% (S)  Infusion related reactions were reported in 1 patient in each treatment group | No outcomes reported |

**References**

1. Yoo DH, Prodanovic N, Jaworski J, Miranda P, Ramiterre E, Lanzon A et al. Efficacy and safety of CT-P13 (biosimilar infliximab) in patients with rheumatoid arthritis: comparison between switching from reference infliximab to CT-P13 and continuing CT-P13 in the PLANETRA extension study. Ann Rheum Dis. 2017;76:355-63. doi:10.1136/annrheumdis-2015-208786.

2. Yoo DH, Prodanovic N, Jaworski J, Miranda P, Ramiterre E, Lanzon A et al. Efficacy and Safety of CT-P13 (Infliximab biosimilar) over Two Years in Patients with Rheumatoid Arthritis: Comparison Between Continued CT-P13 and Switching from Infliximab to CT-P13. American College of Rheumatology; San Diego, California, USA: 2013.

3. Park W, Miranda P, Brzosko M, Wiland P, Gutierrez-Ureña S, Mikazane H et al. Efficacy and Safety of CT-P13 (Infliximab Biosimilar) over Two Years in Patients with Ankylosing Spondylitis: Comparison Between Continuing with CT-P13 and Switching from Infliximab to CT-P13. American College of Rheumatology; San Diego, California, USA: 2013.

4. Park W, Yoo DH, Miranda P, Brzosko M, Wiland P, Gutierrez-Ureña S et al. Efficacy and safety of switching from reference infliximab to CT-P13 compared with maintenance of CT-P13 in ankylosing spondylitis: 102-week data from the PLANETAS extension study. Ann Rheum Dis. 2017;76:346-54. doi:10.1136/annrheumdis-2015-208783.

5. Tanaka Y, Yamanaka H, Takeuchi T, Inoue M, Saito K, Saeki Y et al. Safety and efficacy of CT-P13 in Japanese patients with rheumatoid arthritis in an extension phase or after switching from infliximab. Mod Rheumatol. 2016:1-10. doi:10.1080/14397595.2016.1206244.

6. Yazici Y, Xie L, Ogbomo A, Parenti D, Goyal K, Teeple A et al. A Descriptive Analysis of Real-World Treatment Patterns in a Turkish Rheumatology Population That Continued Innovator Infliximab (Remicade) Therapy or Switched to Biosimilar Infliximab. American College of Rheumatology; Washington DC, USA: 2016.

7. Glintborg B, Kringelbach T, Høgdall E, Sørensen IJ, Jensen DV, Loft AG et al. Non-medical switch from originator to biosimilar infliximab among patients with inflammatory rheumatic disease – impact on s-infliximab and anti-drug antibodies. Results from the national Danish Rheumatologic Biobank and the DANBIO registry. European League Against Rheumatism; London, UK; Ann Rheum Dis: 2016.

8. Glintborg B, Kringelbach T, Høgdall E, Sørensen IJ, Jensen DV, Loft AG et al. Non-medical switch from originator to biosimilar infliximab in patients with inflammatory arthritis: impact on s-infliximab and antidrug antibodies. Results from the Danish Rheumatological Biobank and the DANBIO registry. Scandinavian Congress of Rheumatology; Reykjavik, Iceland: 2016.

9. Glintborg B, Kringelbach TM, Høgdall E, Sørensen IJ, Jensen DV, Loft AG et al. Non-Medical Switch from Originator to Biosimilar Infliximab in Patients with Inflammatory Arthritis – Impact on s-Infliximab and Antidrug-Antibodies. Results from the Danish Rheumatologic Biobank and the Danbio Registry. American College of Rheumatology; Washington DC, USA: 2016.

10. Glintborg B, Sørensen IJ, Jensen DV, Krogh NS, Loft AG, Colic A et al. Three months’ clinical outcomes from a nationwide non-medical switch from originator to biosimilar infliximab in patients with inflammatory arthritis. Results from the DANBIO registry. Scandinavian Congress of Rheumatology; Reykjavik, Iceland: 2016.

11. Glintborg B, Sørensen IJ, Jensen DV, Krogh NS, Loft AG, Espesen J et al. A Nationwide Non-Medical Switch from Originator to Biosimilar Infliximab in Patients with Inflammatory Arthritis. Eleven Months’ Clinical Outcomes from the Danbio Registry. American College of Rheumatology; Washington DC, USA: 2016.

12. Holroyd C, Parker L, Bennett S, Zarroug J, Underhill C, Davidson B et al. Switching to biosimilar infliximab: real-world data from the Southampton biologic therapies review service. British Society for Rheumatology - 2016 Annual Meeting; Glasgow, UK; Rheumatology (Oxford): 2016.

13. Benucci M, Gobbi FL, Bandinelli F, Damiani A, Infantino M, Grossi V et al. Safety, efficacy and immunogenicity of switching from innovator to biosimilar infliximab in patients with spondyloarthritis: a 6-month real-life observational study. Immunol Res. 2016:1-4. doi:10.1007/s12026-016-8843-5.

14. Batticciotto A, Parisi S, Gobbi FL, Antivalle M, Benucci M, Fusaro E et al. Safety and efficacy of switching from innovator to biosimilar infliximab in patients affected by spondyloarthritis. A 6-month observational study. European League Against Rheumatism; London, UK; Ann Rheum Dis: 2016.

15. Batticciotto A, Antivalle M, Gobbi FL, Parisi S, Talotta R, Varisco V et al. Safety and Effcacy of Switching from Originator to CT-P13 Infliximab Biosimilar in Patients Affected By Spondyloarthritis. A 6-Month Observational Study. American College of Rheumatology; Washington DC, USA: 2016.

16. Sokka T, Kautiainen H. Clinical experience with infliximab biosimilar – switch from Remicade. European League Against Rheumatism; Rome, Italy; Ann Rheum Dis: 2015.

17. Nikiphorou E, Kautiainen H, Hannonen P, Asikainen J, Kokko A, Rannio T et al. Clinical effectiveness of CT-P13 (Infliximab biosimilar) used as a switch from Remicade (infliximab) in patients with established rheumatic disease. Report of clinical experience based on prospective observational data. Expert Opin Biol Ther. 2015;15:1677-83. doi:10.1517/14712598.2015.1103733.

18. Abdalla A, Byrne NE, Conway R, Walsh T, Mannion G, Hanly M et al. Long term safety and efficacy of biosimilar infliximab among patients with inflammatory arthritis switched from reference product. European League Against Rheumatism; London, UK; Ann Rheum Dis: 2016.

19. Malaiya R, McKee Z, Kiely P. Infliximab biosimilars—switching Remicade to Remsima in routine care: patient acceptability and early outcome data. British Society for Rheumatology Annual Meeting; Glasgow, UK; Rheumatology (Oxford): 2016.

20. Tweehuysen L, van den Bemt BJF, van Ingen IL, de Jong AJL, van der Laan WH, van den Hoogen FHJ et al. Clinical and Immunogenicity Outcomes after Switching Treatment from Innovator Infliximab to Biosimilar Infliximab in Rheumatic Diseases in Daily Clinical Practice. American College of Rheumatology; Washington DC, USA: 2016.

21. Buer LC, Moum BA, Cvancarova M, Warren DJ, Medhus AW, Hoivik ML. Switching from Remicade(R) to Remsima(R) is safe and feasible: a prospective, open-label study. J Crohns Colitis. 2016 [Epub ahead of print]. doi:10.1093/ecco-jcc/jjw166.

22. Bettey M, Downey L, Underhill C, Callaghan J, Rush M, Ahmed I et al. Outcomes of a managed switching programme changing IBD patients established on originator infliximab to biosimilar infliximab. 11th Congress of the European Crohn's and Colitis Organisation; Amsterdam, The Netherlands: 2016.

23. Jung YS, Park DI, Kim YH, Lee JH, Seo PJ, Cheon JH et al. Efficacy and safety of CT-P13, a biosimilar of infliximab, in patients with inflammatory bowel disease: A retrospective multicenter study. J Gastroenterol Hepatol. 2015;30:1705-12. doi:10.1111/jgh.12997.

24. Jung YS, Park DI, Kim YH, Seo PJ, Kim JW, Kang HW. Efficacy and safety of infliximab's biosimilar (REMSIMA) for IBD. 10th Congress of the European Crohn's and Colitis Organisation; Barcelona, Spain; Journal of Crohns and Colitis: 2015.

25. Smits L, Derikx L, Drenth J, de Jong D, van Esch A, Hoentjen F. Elective switching from Remicade® to biosimilar CT-P13 in inflammatory bowel disease patients: a prospective observational cohort study. 11th Congress of the European Crohn's and Colitis Organisation; Amsterdam, The Netherlands: 2016.

26. Smits LJT, Derikx LAAP, de Jong DJ, Boshuizen RS, van Esch AAJ, Drenth JPH et al. Clinical outcomes following a switch from Remicade® to the biosimilar CT-P13 in inflammatory bowel disease patients: a prospective observational cohort study. J Crohns Colitis. 2016;10:1287-93. doi:10.1093/ecco-jcc/jjw087.

27. Guerra Veloz MF, Argüelles Arias F, Perea Amarillo R, Castro Laria L, Maldonado Pérez MB, Benítez Roldán A et al. Safety and efficacy of infliximab biosimilar (Remsima©) in Crohn’s disease patients in clinical practice: results after 6 months of treatment. 11th Congress of the European Crohn's and Colitis Organisation; Amsterdam, The Netherlands: 2016.

28. Kolar M, Duricová D, Brotlik M, Hruba V, Machkova N, Mitrova K et al. Switching of patients with inflammatory bowel disease from original infliximab (Remicade®) to biosimilar infliximab (Remsima™) is effective and safe. 11th Congress of the European Crohn's and Colitis Organisation; Amsterdam, The Netherlands: 2016.

29. Kolar M, Duricová D, Bortlik M, Hruba V, Machkova N, Mitrova K et al. Switching of patients with inflammatory bowel disease from original infliximab (Remicade®) to biosimilar infliximab (Remsima™) is effective and safe – one-year follow-up. United European Gastroenterology Week; Vienna, Austria; United European Gastroenterology Journal: 2016.

30. Guerra Veloz MF, Argüelles Arias F, Perea Amarillo R, Castro Laria L, Maldonado Pérez MB, Benítez Roldán A et al. Safety and efficacy of infliximab biosimilar (Remsima©) in ulcerative colitis disease patients in clinical practice: results after 6-months treatment. 11th Congress of the European Crohn's and Colitis Organisation; Amsterdam, The Netherlands: 2016.

31. Jarzebicka D, Banaszkiewicz A, Plocek A, Sieczkowska J, Gawronska A, Toporowska-Kowalska E et al. Preliminary assessment of efficacy and safety of switching between originator and biosimilar infliximab in paediatric Crohn disease patients. 10th Congress of the European Crohn’s and Colitis Organisation; Barcelona, Spain: 2015.

32. Kierkus J. Preliminary Assessment of Efficacy and Safety of Switching Between Originator and Biosimilar Infliximab in Paediatric Crohn Disease Patients. 46th Annual Digestive Disease Week; Washington DC, USA; Gastroenterology: 2015.

33. Sieczkowska J, Jarzebicka D, Banaszkiewicz A, Plocek A, Gawronska A, Toporowska-Kowalska E et al. Switching Between Infliximab Originator and Biosimilar in Paediatric Patients with Inflammatory Bowel Disease. Preliminary Observations. J Crohns Colitis. 2016;10:127-32. doi:10.1093/ecco-jcc/jjv233.

34. Sieczkowska J, Jarzebicka D, Oracz G, Meglicka M, Dadalski M, Kierkus J. Immunogenicity after switching from reference infliximab to biosimilar in children with Crohn’s disease. 11th Congress of the European Crohn's and Colitis Organisation; Amsterdam, The Netherlands: 2016.

35. Hlavaty T, Krajcovicova A, Sturdik I, Letkovsky J, Koller T, Toth J et al. Biosimilar infliximab CT-P13 treatment in patients with inflammatory bowel diseases: a 1-year, single-centre retrospective study. 11th Congress of the European Crohn's and Colitis Organisation; Amsterdam, The Netherlands: 2016.

36. Kang HW, Lim YJ, Kim JH, Kang YS. An experience of anti-TNF biosimilar, CT-P13 use: clinical efficacy, safety and interchangeability in inflammatory bowel disease; a pilot study. 9th Congress of the European Crohn’s and Colitis Organisation; Copenhagen, Denmark; J Crohns Colitis: 2014.

37. Kang YS, Moon HH, Lee SE, Lim YJ, Kang HW. Clinical Experience of the Use of CT-P13, a Biosimilar to Infliximab in Patients with Inflammatory Bowel Disease: A Case Series. Dig Dis Sci. 2015;60:951-6. doi:10.1007/s10620-014-3392-z.

38. Armuzzi A. What future for biosimilars in inflammatory bowel diseases? The Italian Group for the study of Inflammatory Bowel Disease VII National Congress; Palermo, Italy: 2015.

39. Fiorino G, Manetti N, Variola A, Bossa F, Rizzuto G, Guidi L et al. Prospective observational study on inflammatory bowel disease patients treated with infliximab biosimilars: preliminary results of the PROSIT-BIO cohort of the IG-IBD. 11th Congress of the European Crohn's and Colitis Organisation; Amsterdam, The Netherlands: 2016.

40. Park SH, Kim YH, Lee JH, Kwon HJ, Lee SH, Park DI et al. Post-marketing study of biosimilar infliximab (CT-P13) to evaluate its safety and efficacy in Korea. Expert Rev Gastroenterol Hepatol. 2015;9 Suppl 1:35-44. doi:10.1586/17474124.2015.1091309.

41. Dapavo PMD, Vujic IMD, Fierro MTMD, Quaglino PMD, Sanlorenzo MMD. The infliximab biosimilar in the treatment of moderate to severe plaque psoriasis. J Am Acad Dermatol. 2016;75:736-9. doi:10.1016/j.jaad.2016.04.068.

42. Jørgensen K, Olsen I, Goll G, Lorentzen M, Bolstad N, Haavardsholm E et al. Biosimilar infliximab (CT-P13) is not inferior to originator Infliximab: results from the 52-week randomized NOR-SWITCH trial. United European Gastroenterology Week; Vienna, Austria; United European Gastroenterology Journal: 2016.

43. Goll GL, Olsen IC, Jorgensen KK, Lorentzen M, Bolstad N, Haavardsholm EA et al. Biosimilar Infliximab (CT-P13) Is Not Inferior to Originator Infliximab: Results from a 52-Week Randomized Switch Trial in Norway. American College of Rheumatology; Washington DC, USA; Arthritis Rheumatol: 2016.

44. Gentileschi S, Barreca C, Bellisai F, Biasi G, Brizi MG, De Stefano R et al. Switch from infliximab to infliximab biosimilar: efficacy and safety in a cohort of patients with different rheumatic diseases: Response to: Nikiphorou E, Kautiainen H, Hannonen P, et al. Clinical effectiveness of CT-P13 (Infliximab biosimilar) used as a switch from Remicade (infliximab) in patients with established rheumatic disease. Report of clinical experience based on prospective observational data. Expert Opin Biol Ther. 2015;15:1677–1683. Expert Opin Biol Ther. 2016;16:1311-2. doi:10.1080/14712598.2016.1198765.

45. Smolen J, Choe JY, Prodanovic N, Niebrzydowski J, Staykov I, Dokoupilova E et al. Comparable safety and immunogenicity and sustained efficacy after transition to SB2 (an infliximab biosimilar) vs ongoing infliximab reference product in patients with rheumatoid arthritis: results of phase III transition study. EULAR16 Abstract FRI0162: 2016.

46. Smolen JS, Choe JY, Prodanovic N, Niebrzydowski J, Staykov I, Dokoupilova E et al. Comparable Safety and Immunogenicity and Sustained Efficacy after Transition to SB2 (An Infliximab Biosimilar) Vs Ongoing Reference Infliximab (Remicade®) in Patients with Rheumatoid Arthritis: Results of Phase III Transition Study. American College of Rheumatology; Washington DC, USA: 2016.

47. Hamanaka S, Nakagawa T, Koseki H, Sakurai T, Taida T, Okimoto K et al. Infliximab biosimilar in the treatment of inflammatory bowel disease: a Japanese single-cohort observational study. 11th Congress of the European Crohn's and Colitis Organisation; Amsterdam, The Netherlands: 2016.

48. Díaz Hernández L, Rodríguez González GE, Vela González M, Tardillo Marín CA, Rodríguez Díaz CY, Arranz Hernández L et al. Efficacy and safety of switching between originator and biosimilar infliximab in patietns with inflammatory bowel disease in practical clinic: results to 6 months. 11th Congress of the European Crohn's and Colitis Organisation; Amsterdam, The Netherlands: 2016.

49. Emery P, Vencovský J, Sylwestrzak A, Leszczynski P, Porawska W, Stasiuk B et al. Longterm safety and efficacy of SB4 (etanercept biosimilar) in patients with rheumatoid arthritis: comparison between continuing SB4 and switching from etanercept reference product to SB4. European League Against Rheumatism; London, UK; Ann Rheum Dis: 2016.

50. Emery P, Vencovský J, Sylwestrzak A, Leszczynski P, Porawska W, Stasiuk B et al. Additional Efficacy Results of SB4 (Etanercept Biosimilar) up to Week 100: Comparison Between Continuing SB4 and Switching from Reference Etanercept (Enbrel®) to SB4. American College of Rheumatology; Washington DC, USA: 2016.

51. Lee Y, Shin D, Kim Y, Kang J, Gauliard A, Fuhr R. A randomised Phase l pharmacokinetic study comparing SB4 and etanercept reference product (Enbrel®) in healthy subjects. Br J Clin Pharmacol. 2016;82:64-73. doi:10.1111/bcp.12929.

52. Lee YJ, Shin D, Kim Y, Kang JW, Fuhr R, Gauliard A. A phase I pharmacokinetic study comparing SB4, an etanercept biosimilar, and etanercept reference product (Enbrel®) in healthy male subjects. European League Against Rheumatism; Rome, Italy; Ann Rheum Dis: 2015.

53. Griffiths CE, Thaci D, Gerdes S, Arenberger P, Pulka G, Kingo K et al. The EGALITY study: A confirmatory, randomised, double-blind study comparing the efficacy, safety and immunogenicity of GP2015, a proposed etanercept biosimilar, versus the originator product in patients with moderate to severe chronic plaque-type psoriasis. Br J Dermatol. 2016 [Epub ahead of print]. doi:10.1111/bjd.15152.

54. Afonso M, Sanguino Heinrich S, Poetzl J, Woehling H. Pharmacokinetics and safety of GP2015, a proposed etanercept biosimilar, and etanercept originator product in healthy male subjects: a randomised twoway crossover study. European League Against Rheumatism; London, UK; Ann Rheum Dis: 2016.

55. Cohen S, Pablos JL, Zhang N, Rizzo W, Muller G, Padmanaban D et al. ABP 501 Long-Term Safety/Efficacy: Interim Results from an Open-Label Extension Study. American College of Rheumatology; Washington DC, USA: 2016.

56. Strober B, Foley P, Kaur P, Philipp S, Zhang N. Evaluation of Efficacy and Safety of ABP 501 in a Phase 3 Study in Subjects with Moderate to Severe Plaque Psoriasis: 52-week results. American Academy of Dermatology 74th Annual Meeting; Washington DC, USA; J Am Acad Dermatol: 2016.

57. Gooderham M, Constanzo A, Kaliaperumal A, Kaur P, Narbutt J, Spelman L et al. Single Transition from Adalimumab to ABP 501: Evaluation of Immunogenicity in a Phase 3 Study in Subjects with Moderate to Severe Plaque Psoriasis. American Academy of Dermatology 74th Annual Meeting; Washington DC, USA; J Am Acad Dermatol: 2016.

58. Weinblatt M, Baranauskaite A, Niebrzydowski J, Dokoupilova E, Zielinska A, Sitek-Ziolkowska K et al. Sustained efficacy and comparable safety and immunogenicity after transition to SB5 (an adalimumab biosimilar) vs continuation of the adalimumab reference product in patients with rheumatoid arthritis: result of Phase III study. European League Against Rheumatism; London, UK; Ann Rheum Dis: 2016.

59. Genovese MC, Weinblatt M, Keystone EC, Baranauskaite A, Cheong SY, Ghil J. Efficacy after Transition to SB5 from Reference Adalimumab (Humira®) Vs. Continuation of SB5 or Reference Adalimumab By Antibodies Developed after Transition from a SB5 Phase III Study. American College of Rheumatology; Washington DC, USA: 2016.

60. Yoo DH, Park W, Suh CH, Shim SC, Cons Molina F, Jeka S et al. Efficacy and Safety of Switched CT-P10 from Innovator Rituximab Compared to Those of Maintained CT-P10 in Patients with Rheumatoid Arthritis up to 56 Weeks. American College of Rheumatology; San Francisco, California, USA; Arthritis Rheumatol: 2015.
